# Evolution of a transcriptional regulator from a transmembrane nucleoporin

**DOI:** 10.1101/gad.280941.116

**Published:** 2016-05-15

**Authors:** Tobias M. Franks, Chris Benner, Iñigo Narvaiza, Maria C.N. Marchetto, Janet M. Young, Harmit S. Malik, Fred H. Gage, Martin W. Hetzer

**Affiliations:** 1 Laboratory of Molecular and Cellular Biology, Salk Institute for Biological Studies, La Jolla, California 92037, USA;; 2 Laboratory of Genetics, Salk Institute for Biological Studies, La Jolla, California 92037, USA;; 3 Basic Sciences Division, Fred Hutchinson Cancer Research Center, Seattle, Washington 98109, USA;; 4 Howard Hughes Medical Institute, Fred Hutchinson Cancer Research Center, Seattle, Washington 98109, USA;; 5 Center for Academic Research and Training in Anthropogeny (CARTA), La Jolla, California 92093, USA

**Keywords:** evolution, hominoid, Pom121, Nup98, transcription, nuclear pore complex (NPC)

## Abstract

Nuclear pore complexes (NPCs) emerged as nuclear transport channels in eukaryotic cells ∼1.5 billion years ago. While the primary role of NPCs is to regulate nucleo–cytoplasmic transport, recent research suggests that certain NPC proteins have additionally acquired the role of affecting gene expression at the nuclear periphery and in the nucleoplasm in metazoans. Here we identify a widely expressed variant of the transmembrane nucleoporin (Nup) *Pom121* (named *sPom121*, for “*soluble Pom121*”) that arose by genomic rearrangement before the divergence of hominoids. *sPom121* lacks the nuclear membrane-anchoring domain and thus does not localize to the NPC. Instead, *sPom121* colocalizes and interacts with nucleoplasmic *Nup98*, a previously identified transcriptional regulator, at gene promoters to control transcription of its target genes in human cells. Interestingly, *sPom121* transcripts appear independently in several mammalian species, suggesting convergent innovation of Nup-mediated transcription regulation during mammalian evolution. Our findings implicate alternate transcription initiation as a mechanism to increase the functional diversity of NPC components.

## Abstract (precis)

Franks et al. identify a widely expressed variant of the transmembrane nucleoporin *Pom121* (named *sPom121*, for “soluble *Pom121*”) that arose by genomic rearrangement before the divergence of hominoids. Instead of localizing to the NPC, *sPom121* colocalizes and interacts with nucleoplasmic *Nup98*, a previously identified transcriptional regulator, at gene promoters to control transcription of its target genes in human cells.

The nuclear pore complex (NPC) is an intricate assembly of ∼30 different nucleoporins (Nups) that promotes regulated transport of cargo to and from the nucleus (Wente and Rout 2010; Hoelz et al. 2011; Solmaz et al. 2011; Raices and D'Angelo 2012; Hurt and Beck 2015). Although the nucleotide sequences of Nup genes have undergone significant evolutionary changes, the structure of the NPC has remained remarkably well conserved, with essentially no change in protein organization occurring after the appearance of the last eukaryotic common ancestor (LECA) (Devos et al. 2004, 2006; Alber et al. 2007; Brohawn et al. 2008; DeGrasse et al. 2009). In particular, the NPC scaffold Nups, composed of the *Nup107/160* and *Nup93/205* complexes, typically contain α-solenoid and β-propeller domains that share strong structural similarities to proteins that coat transport vesicles (Devos et al. 2004, 2006; Alber et al. 2007; Brohawn et al. 2008). Based on such similarities, the proto-coatomer hypothesis proposed that NPCs and clathrin, COPI, and COPII vesicle coats share a common evolutionary origin in an early membrane-curving module, the “proto-coatomer” (Devos et al. 2004, 2006; Brohawn et al. 2008; Leksa and Schwartz 2010). Other components of the NPC, including the nuclear basket and cytoplasmic filaments, are, for the most part, also well conserved throughout eukaryotes (Field et al. 2014). In contrast, the transmembrane (TM) components of the NPC are often lineage-restricted and have undergone dramatic changes from yeast to humans (Neumann et al. 2010; Field et al. 2014). This is best exemplified by the recent appearance of *Pom121*, which emerged in vertebrates, where it has an essential role in interphase NPC assembly (Doucet et al. 2010; Dultz and Ellenberg 2010; Funakoshi et al. 2011; Talamas and Hetzer 2011; Field et al. 2014).

Although the composition of the NPC is highly conserved, the functional repertoire of many Nups has expanded throughout evolutionary history. One possible driver of this functional diversity might be the NPC's remarkable ability to act as a scaffold for protein assemblies involved in diverse cellular processes, including the regulation of the cell cycle, DNA damage response, and transcription regulation (Raices and D'Angelo 2012; Burns and Wente 2014; Ptak et al. 2014; Ibarra and Hetzer 2015). One potential limitation for the NPC in regulating processes such as transcription is the strict localization of Nups to the NPC at the nuclear envelope (NE), which limits the number of genes that can be regulated to those that are in close proximity to the nuclear periphery. For example, in yeast, gene regulation by the NPC is most likely confined to a small subset of genes that are targeted to active transcriptional regions around NPCs to promote gene induction or those that are organized in gene loops, which stimulate rapid transcriptional reinitiation of genes following a period of suppression (Brickner and Walter 2004; Casolari et al. 2004; O'Sullivan et al. 2004; Cabal et al. 2006; Taddei et al. 2006; Tan-Wong et al. 2009; Brickner and Brickner 2011). Thus, in order to increase the number of transcriptional targets that can potentially be regulated by Nups, it would be necessary to uncouple the specific functions of Nups in NPC-mediated transport from those in gene regulation. Consistent with such a notion, previous research in human cells demonstrated that a subset of peripheral Nups can move on and off human NPCs, raising the possibility that Nups’ influence on gene expression could extend beyond genomic regions associated with the NE (Rabut et al. 2004). Supporting this hypothesis, the dynamic movement of a subset of Nups, including *Nup50*, *Nup98*, and *Nup153*, was slowed in the presence of transcriptional inhibitory drugs, suggesting a role for *Nup98* and potentially other Nups in the regulation of transcription (Griffis et al. 2002, 2004; Buchwalter et al. 2014). A breakthrough in our understanding of the function of mobile Nups came from studies in *Drosophila* and mammalian cells, which determined that *Nup98* and several other peripheral Nups such as *Nup50*, *Nup62*, and *Nup153* can detach from the NPC, bind to intranuclear promoters distal to the NE, and affect regulation of adjacent genes (Capelson et al. 2010; Kalverda and Fornerod 2010; Liang et al. 2013). Although the mechanism of Nup98-mediated gene regulation in the nucleoplasm is yet to be determined in detail, recent evidence suggests that *Nup98* interacts with *CBP/p300* and *MBD-R2/NSL* chromatin-modifying complexes in human and *Drosophila* cells, respectively, suggesting a possible mechanism by which *Nup98* promotes an active chromatin state (Kasper et al. 1999; Pascual-Garcia et al. 2014). Most of the evidence collected in *Drosophila* suggested that only peripheral Nups can exist away from the NPC (Capelson et al. 2010). However, a soluble fraction of the *Nup107/160* scaffold complex was recently shown to colocalize with *Nup98* in foci in the nucleoplasm of human HeLa cells (Morchoisne-Bolhy et al. 2015).

The finding of bifunctional Nups, which mediate transport at the NPC and transcription in the nucleoplasm, suggests that significant changes might have occurred during evolution to increase functionality without disrupting an existing essential function. Nevertheless, these two functions suggest the possibility of pleiotropy, in which optimal functionality for both transport and transcription functions cannot be accommodated within a single protein-coding gene (Guillaume and Otto 2012), reducing the adaptive capacity of the NPC (Orr 2000). Gene duplication has been proposed as one mechanism that can relieve the deleterious effects of such antagonistic pleiotropy of functions under certain conditions (Guillaume and Otto 2012). However, the possibility of such subfunctionalization in the NPC has not been previously explored.

Here, we identify a variant of the TM Nup gene *POM121* that produces a soluble (i.e., non-membrane-bound) form of *Pom121* (*sPom121*) that lacks its TM domain and is no longer incorporated into the NPC. In hominoids, the *sPom121* transcript is expressed from an alternative transcriptional start site that arose from a genomic rearrangement. This novel isoform includes new 5′ untranslated region (UTR) exons and bypasses the canonical TM-coding exon to encode an N-terminally truncated form of *Pom121*. Functionally, *sPom121* and *Nup98* cobind specific gene promoters to regulate transcription in human cells. Thus, *sPom121* represents the first validated example of an NPC component that has eschewed its role in nucleo–cytoplasmic transport to specialize in an unrelated process; namely, gene regulation. In addition, we show that *sPom121* can promote retention of *Nup107/160* complexes in the nucleoplasm during NPC formation, suggesting that the evolution of *sPom121* brought about dramatic functional expansion of other scaffold Nups in hominoid cells.

## Results

### Alternative transcription initiation produces a sPom121 isoform in humans

**Figure 1.**
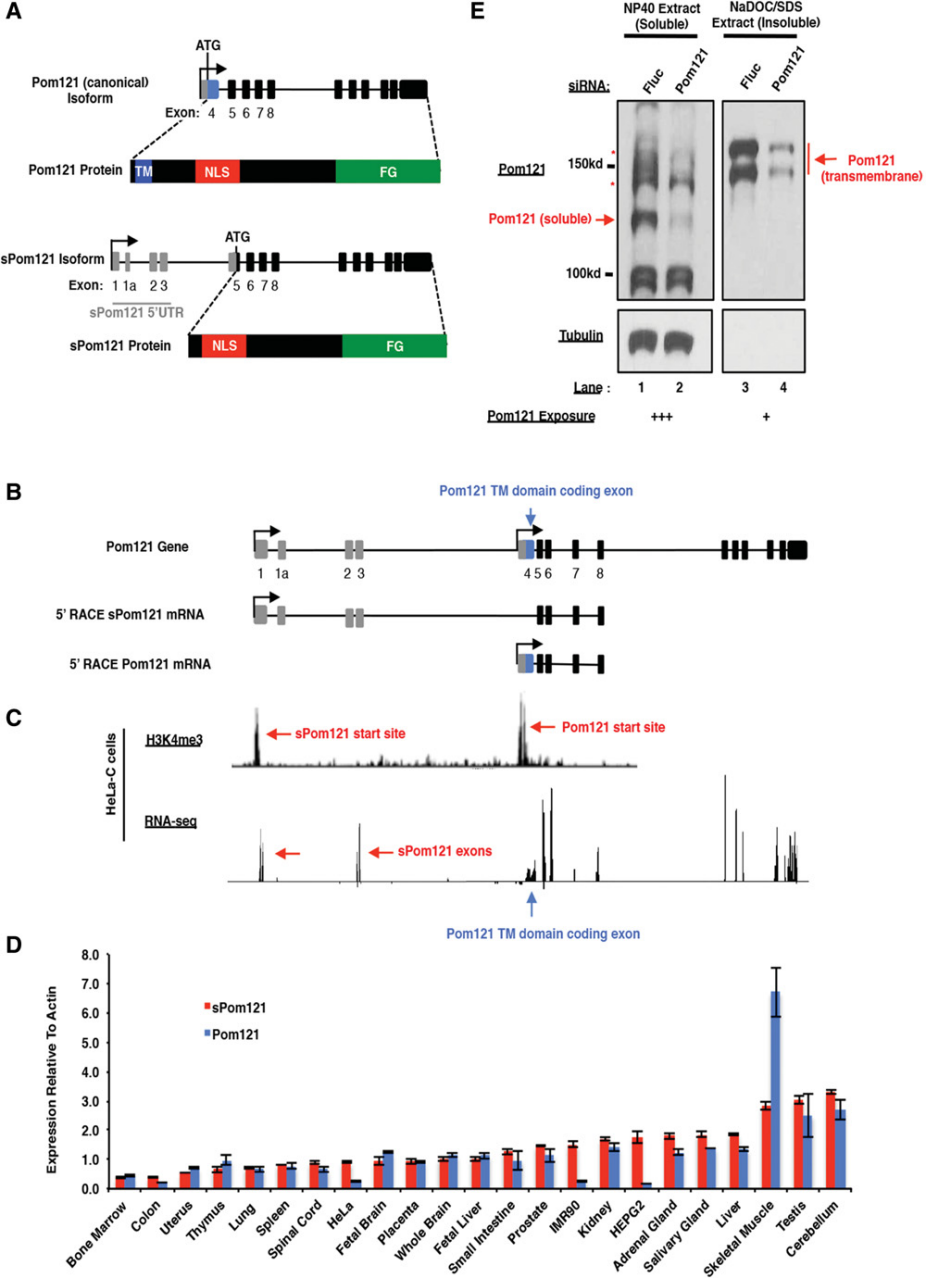
Detection of sPom121 mRNA and protein expression in human cells. (*A*) Schematic of two putative Pom121 isoforms expressed in humans and confirmed by 5′ rapid amplification of cDNA ends (RACE). (*Top*) Gray boxes indicate 5′ UTR-encoding exons, blue boxes indicate TM domain-encoding exon, and black boxes indicate Pom121-encoding exons. (*Bottom*) The TM domain, nuclear localization signal (NLS), and phenylalanine/glycine (FG) domain are indicated (blue, red, and green, respectively). (*B*) Schematic showing the annotated 5′ end of the Pom121 gene (shown at the *top*, “Pom121 gene”) compared with that of sPom121 (“5′ RACE sPom121 mRNA”) and Pom121 (“5′ RACE Pom121 mRNA”), identified here by 5′ RACE. (*C*) Histone H3 Lys4 trimethylation (H3K4me3) (*top*) and RNA sequencing (RNA-seq) (*bottom*) results from HeLa-C cells. Red arrows are used to indicate active transcriptional start sites (*top*), sPom121-specific exons (*bottom left*), or the TM-coding exon of Pom121 (*bottom right*). (*D*) sPom121 and Pom121 expression in various tissues. Quantitative PCR (qPCR) analysis of sPom121 (red bars) and Pom121 (blue bars) mRNA levels in multiple tissue types relative to actin. Results are plotted such that the tissue with the lowest sPom121 mRNA expression is at the *left*, while the tissue expressing the highest levels of sPom121 is shown at the *right*. Different primers were used to analyze sPom121 and Pom121 cDNA levels, and thus a comparison of sPom121 and Pom121 levels in each tissue cannot be made from these data. (*E*) Western blot to detect sPom121. Soluble (lanes *1*,*2*) and insoluble (lanes *3*,*4*) lysates were electrophoresed and Western blotted, and proteins were detected with a Pom121 antibody (*top* panels) or tubulin (*bottom* panels). (Lanes *2*,*4*) Samples that had been treated with Pom121 siRNA are included to identify which bands correspond to Pom121 protein. Pom121 blots were exposed for 30 sec (*left* blot) or 10 sec (*right* blot).

While investigating expressed sequence tags (ESTs) for human *Pom121* transcripts, we noticed, consistent with previous reports (Funakoshi et al. 2007), that there is an abundance of sequences that contain a noncanonical 5′ UTR sequence and lack the TM-coding sequence of Pom121 (Fig. 1A, “*sPom121* isoform”). These noncanonical transcripts, here called the *sPom121* mRNA, are predicted to initiate at an alternative transcription start site ∼40 kb upstream of the TM-encoding canonical first exon (here called exon 4), include three or four exons in this upstream region, and splice past the exon 4 to the second “canonical” coding exon of *Pom121* (exon 5) (Fig. 1A). As a result, we predict that *sPom121* transcripts would be translated beginning at an ATG codon in exon 5, encoding an N-terminally truncated form of *Pom121* that is missing the N-terminal TM domain.

To explore the expression of the *sPom121* mRNA in human cells and confirm the *sPom121* gene model, we conducted 5′ RACE (rapid amplification of cDNA ends) experiments in six cell lines: HeLa-C, HeLa, U2OS, IMR90, RPE-1, and SJSA. Importantly, a reverse oligo was chosen that anneals downstream from the *Pom121* TM-coding region at a site that is predicted to be shared between *Pom121* and *sPom121* transcripts. The amplified DNA products migrate as a smear at ∼1500 base pairs (bp) (Supplemental Fig. S1A). Cloning and sequencing revealed that most *Pom121* 5′ sequences (22 of 24 HeLa-C clones, four of five U2OS clones, and four of four IMR90 clones) corresponded to the predicted *sPom121* transcript, which lacks a TM-coding region (Fig. 1B; Supplemental Fig. S1B,D). RACE analysis further revealed that this transcript contains three or four additional exons unique to the *sPom121* mRNA (Fig. 1B). In contrast, the other *Pom121* transcripts (three of 33 clones for HeLa, U2OS, and IMR90 combined) corresponded to the canonical *Pom121* mRNA, which has a short 5′ UTR and includes the *Pom121* TM domain (Fig. 1B; Supplemental Fig. S1C,D). These results demonstrated that *sPom121* and *Pom121* mRNAs are the products of two different transcriptional start sites.

To further confirm alternate transcriptional initiation, we conducted chromatin immunoprecipitation (ChIP) experiments in HeLa-C cells with antibodies raised against histone H3 Lys4 trimethylation (H3K4me3), a histone mark that is a reliable indicator of active promoters, followed by ChIP sequencing (ChIP-seq) experiments. As shown in Figure 1C, H3K4me3 peaks are clearly observed at both the previously identified *Pom121* transcriptional start site and an additional upstream start site that corresponds to the 5′ end of the *sPom121* RACE product (identified by red arrows). RNA sequencing (RNA-seq) analyses in HeLa-C cells further confirmed the gene model for *sPom121*, including the newly identified first three exons (Fig. 1C, red left-facing arrows). In addition to the *sPom121*-unique exons, reads corresponding to exon 4 (Fig. 1C, blue up-facing arrow) can be used to unambiguously analyze the expression of the canonical *Pom121* mRNA. The unique exons of *sPom121* were a useful tool to allow us to determine whether *sPom121* expression is consistent across all cell types or is tissue-specific. To this end, we synthesized cDNA and conducted quantitative PCR (qPCR) to quantify *sPom121* and *Pom121* expression levels from a panel of 20 different human tissues. We found that expression of both *sPom121* and *Pom121* mRNA is widely expressed across tissues, although at varying levels; the highest *sPom121* expression is observed in the cerebellum, testis, skeletal muscle, and liver (Fig. 1D; Supplemental Fig. S1E,F).

We next tested whether the *sPom121* mRNA produces a functional protein. Using Western blotting, we attempted to unequivocally determine the presence of a soluble, TM-lacking form of *Pom121*. We first sequentially extracted soluble and then membrane-bound proteins from cells that had been treated with either a control siRNA targeting firefly luciferase (Fluc) or an siRNA that targets a sequence that is shared between *Pom121* and *sPom121*. The lysates were separated by SDS-PAGE and analyzed using specific anti-*Pom121* antibodies. We detected a band of ∼125 kDa (Fig. 1E, lane 1, red arrow) in the soluble lysate, which correlates with the expected size of a form of *Pom121* lacking its N-terminal TM domain. Importantly, the intensity of the 125-kDa band is strongly reduced upon knockdown of *Pom121* with an siRNA, suggesting that this protein corresponds to endogenous *sPom121*. As expected, full-length *Pom121* (Fig. 1E, lanes 3,4) was observed primarily in the insoluble lysate running as a doublet at the expected size of ∼150 kDa, as would be consistent with a TM domain-containing protein. The ability to extract *Pom121* and *sPom121* using different conditions further suggests that the two proteins are in distinct complexes.

### sPom121 localizes to the nucleoplasm instead of the NPC

**Figure 2.**
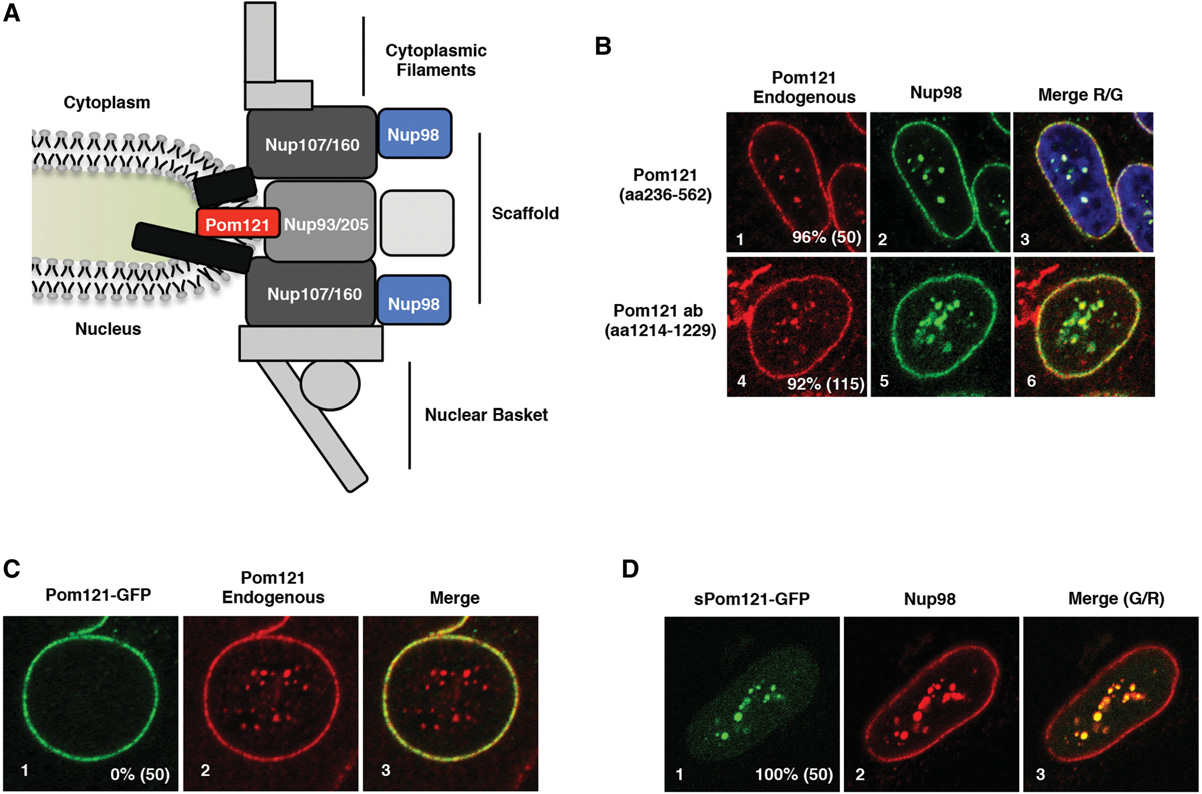
sPom121 protein localizes in the nucleoplasm of human cells. (*A*) Schematic of the NPC components. (Gray, nuclear side of the NE) Cytoplasmic filaments; (gray, cytoplasmic side of the NE) nuclear basket; (black) Nup107/160 complex; (dark gray) Nup93-205 complex; (black with Pom121 highlighted in red) TM Nups; (blue) Nup98. (*B*) IF assays showing localization of endogenous Pom121 (panels *1*,*4*) and Nup98 (panels *2*,*5*) in HeLa-C cells. Merged images are shown in panels *3* and *6*. (*C*) Comparison of localization of transfected rat Pom121-3GFP (panel *1*) with endogenous Pom121 (panel *2*). A merged image is shown in panel *3*. The percentage of cells with intranuclear localization of the Pom121-3GFP is indicated in the *bottom right* corner of panel *1*, while the number of cells quantified is in parentheses. (*D*) Comparison of sPom121-GFP (rat Pom121 amino acids 241–1200) with Nup98 in HeLa-C cells. The percentage of cells with intranuclear localization of the Pom121-3GFP is indicated in the *bottom right* corner of panel *1*, while the number of cells quantified is in parenthesis.

In order to test whether *Pom121* and *sPom121* have distinct subcellular localization, we conducted immunofluorescence (IF) assays in HeLa-C cells with antibodies raised against three different regions of *Pom121*. We specifically used HeLa-C cells because they are characterized by intranuclear Nup foci that facilitate visualization of intranuclear Nups by IF (Xu and Powers 2010; Morchoisne-Bolhy et al. 2015). As expected, we observed *Pom121* localization to the nuclear periphery, consistent with localization to the NPC (Fig. 2A,B). In addition to this nuclear rim localization, we observed a pool of intranuclear *Pom121* colocalizing with Nup98 in the nucleoplasm (Fig. 2B). This intranuclear localization of *sPom121* was confirmed with two additional antibodies that recognize distinct epitopes of the *Pom121* protein, suggesting specificity of the signal for the endogenous protein (Fig. 2B; Supplemental Fig. S2A).

To test whether other Nups are recruited to *sPom121* foci, we compared its localization with that of other Nups, including components of the nuclear basket, cytoplasmic filaments, and scaffold structure (Fig. 2A). We readily observed components of the *Nup107/160* scaffold complex, including *Nup96*, *Nup133*, *Nup107*, and *Elys*, colocalizing with *Nup98* (Supplemental Fig. S2B, panels 2,5,8.11) in the nucleoplasm in >90% of cells, similar to what has been shown previously (Morchoisne-Bolhy et al. 2015). In contrast to *Pom121* and the *Nup107/160* complex, we did not observe nuclear basket components, components of the cytoplasmic filaments, the *Nup93/205* scaffold complex, or any of the basket and cytoplasmic filament Nups detected by antibody mAB414 colocalizing with intranuclear *Nup98* (Supplemental Fig. S2C). These findings suggest that *sPom121*-containing structures do not resemble intranuclear NPCs.

Next, we wondered whether *sPom121* protein is present in other cell types. To test this, we overexpressed GFP-*Nup98* to induce macroscopic *Nup98* foci, which facilitate the detection of potential *sPom121*. We then probed cells with anti-*Pom121* antibodies. Indeed, overexpression of GFP-*Nup98* in HeLa, U2OS, and IMR90 cells induces colocalization of *Pom121* and GFP-*Nup98* in 94%, 94%, and 86% of cells, respectively (Supplemental Fig. S2D). In addition, the *Nup107/160* complex, but not factors detected by mAB414, was also observed to colocalize with intranuclear GFP-*Nup98* in U2OS cells (data not shown).

We considered whether *sPom121* foci in the nucleoplasm could be the result of NE invaginations, which can be observed at relatively low frequency in many cell types. We overexpressed full-length rat *Pom121*-GFP (which contains a TM domain) to test for colocalization with endogenous *Pom121* in the nuclear interior in HeLa-C cells. As expected, *Pom121*-GFP was observed only at the nuclear rim and not colocalizing with endogenous *Pom121* in the nucleoplasm (Fig. 2C). Moreover, we did not observe colocalization of intranuclear *Pom121* foci with all NPC components (see above) or with lamina proteins (data not shown), suggesting that *sPom121* is not associated with NPCs. These results suggest that endogenous *sPom121* likely represents a soluble variant that lacks the N-terminal TM domain of *Pom121*. To confirm this, we cloned and expressed the GFP-tagged *sPom121* (*Pom121* amino acids 241–1200) (Fig. 2D) and conducted IF assays to compare *sPom121*-GFP localization with that of *Nup98*. We found that *sPom121*-GFP (Fig. 2D, panel 1) colocalizes with *Nup98* (Fig. 2D, panel 2) and phenocopied the localization of the endogenous *sPom121* protein (Fig. 2, cf. D [panel 1] and B [panels 1,4]). Finally, our Western blot analyses (Fig. 1D) confirm that *Pom121* and *sPom121* are in distinct complexes, with the latter representing a soluble variant (Fig. 1D). We therefore conclude that *sPom121* colocalizes in the nucleoplasm with *Nup98* and the *Nup107/160* complex in a broad range of human cell types.

### The nuclear localization signal (NLS) domain of sPom121 is required for colocalization with Nup98

To further understand how *sPom121* functions in the nucleoplasm, we wanted to determine how *sPom121* colocalizes with *Nup98* and the *Nup107/160* complex in the nucleoplasm. Previous studies suggest that the NLS domain of *Pom121* is crucial for interactions with various Nups at the NPC, including *Nup98* and the *Nup107/160* complex (Mitchell et al. 2010; Yavuz et al. 2010; Shaulov et al. 2011). In addition, the NLS domain is required for most of *Pom121*'s described roles at the NPC (Yavuz et al. 2010; Shaulov et al. 2011). We therefore hypothesized that, like canonical *Pom121* at the NPC, *sPom121* uses its NLS domain to interact with *Nup98* and the *Nup107/160* complex except it does so in the nucleoplasm.

**Figure 3.**
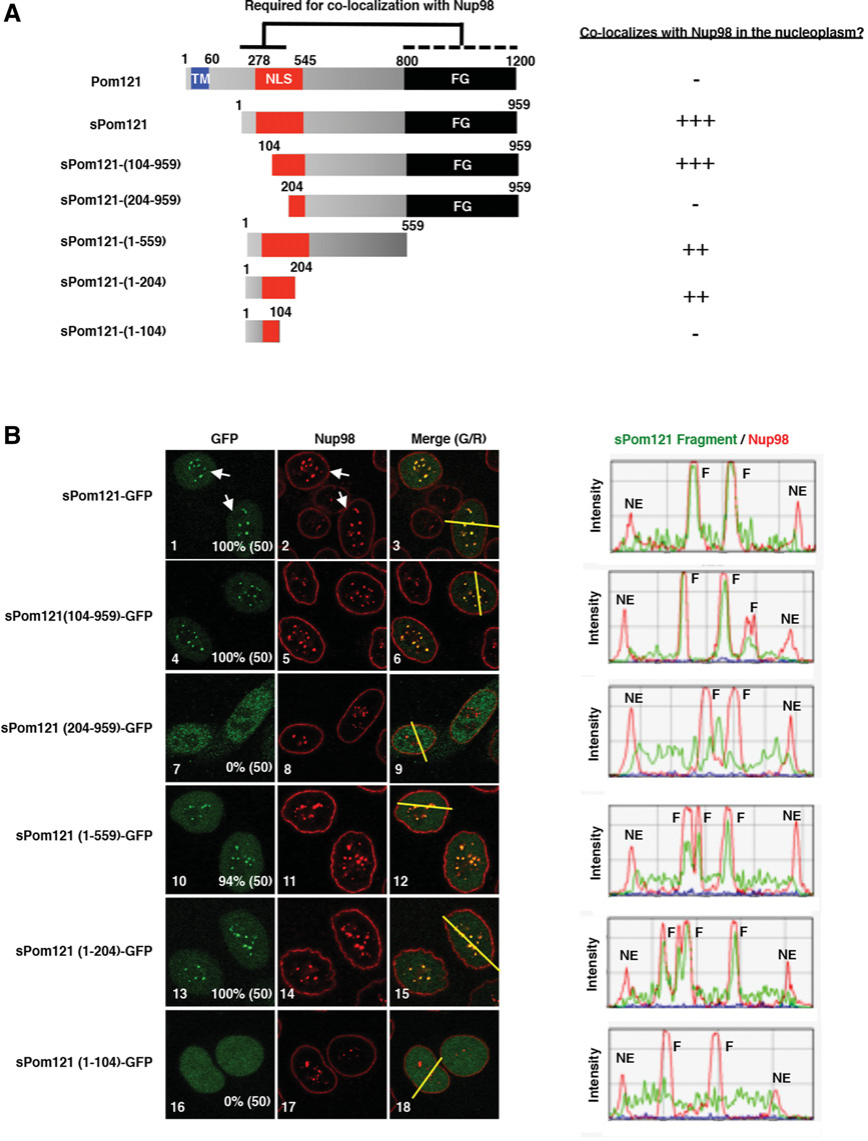
The NLS domain of sPom121 is required for colocalization with Nup98. (*A*) Schematic of Pom121 mutants used to identify the domain required to localize sPom121 to Nup98 foci. All mutants were cloned from a rat Pom121-3GFP construct that was described previously (Doucet et al. 2010; Talamas and Hetzer 2011). The Pom121 TM domain (blue), NLS domain (red), and FG domain (black) are shown. The fragment of sPom121 required for colocalization with Nup98 is indicated with a black bar (*top*), while the region that has a minor effect on sPom121 localization is indicated with a dotted black bar (*top*) (*B*) IF assays showing localization of the sPom121 mutants listed at the *left* (panels *1*,*4*,*7*,*10*,*13*,*16*) and Nup98 (panels *2*,*5*,*8*,*11*,*14*,*17*). Merged images are shown at the *right* (in panels *3*,*6*,*9*,*12*,*15*,*18*). The percentage of cells with the Pom121 mutant colocalizing with GFP-Nup98 in the nucleoplasm is shown in the *bottom right* corner of the *left* panels, while the number of cells counted is shown in parentheses. (Panels *3*,*6*,*9*,*12*,*15*,*18*) The fluorescence intensity of sPom121 and Nup98 at either the nuclear membrane or nucleoplasmic foci was observed by quantifying the intensity of the yellow lines drawn through a cell cross-section. The intensity graphs are shown at the *right*. (F) Focus. sPom121 only colocalizes with Nup98 in nucleoplasmic foci but never at the NE.

To determine which domain of *sPom121* is required for colocalization with *Nup98*, we took advantage of our *sPom121*-GFP construct (Fig. 2D) and created a series of *sPom121* deletion mutants lacking the C-terminal phenylalanine/glycine (FG) domain, the central domain, and portions of the N-terminal NLS domain (Fig. 3A). We assayed both the full-length and deletion *sPom121*-GFP proteins for colocalization with *Nup98* using IF analysis. As shown in Figure 3B, amino acids 1–204 of *sPom121* are necessary and sufficient for *sPom121* colocalization with *Nup98* (cf. panels 13 and 14 and the line graph at the right) in the nucleoplasm, while the FG domain appears to serve a minor role, as demonstrated by an increase in diffuse nuclear localization of *sPom121* in FG domain mutants (cf. panels 10,11 and 1,2 and see the line graphs at the right). We also observed a marked increase in the size and intensity of *Nup98* foci in cells expressing *sPom121*-GFP (Fig. 3B, panel 2, white arrows), suggesting that *sPom121* overexpression can promote the retention of *Nup98* and potentially other Nups such as the *Nup107/160* complex in intranuclear bodies. We conclude that the NLS domain of *sPom121* is required for colocalization with *Nup98* and the *Nup107/160* complex, while the FG domain of *sPom121* plays a secondary role in enhancing the affinity of *sPom121* for *Nup98* complexes.

### sPom121 and Nup98 have similar intranuclear dynamics

**Figure 4.**
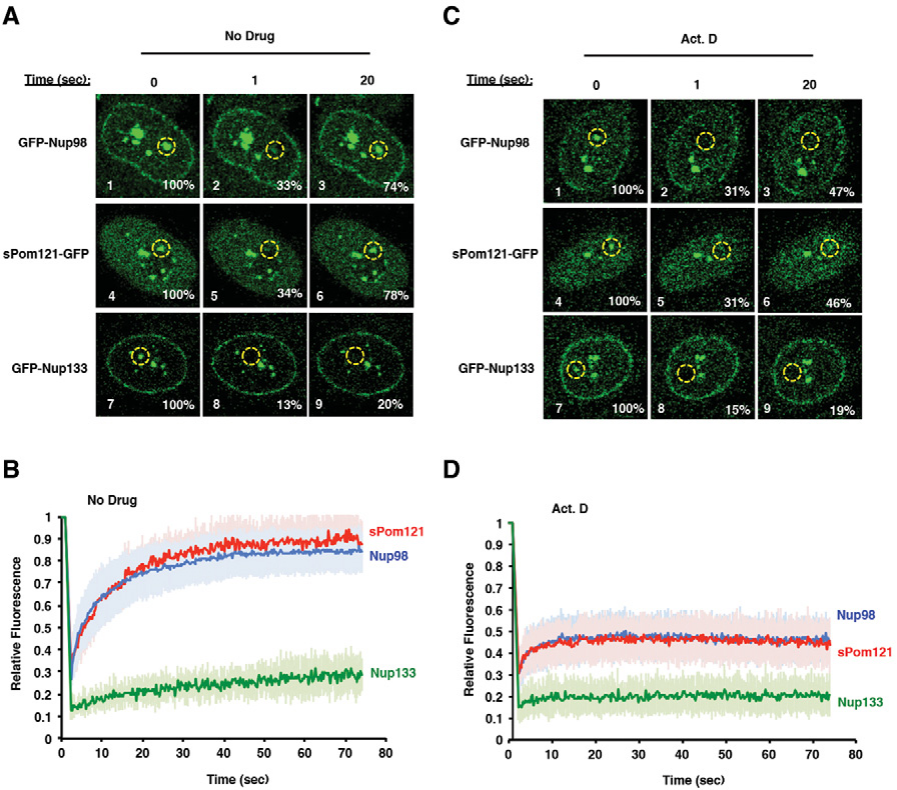
sPom121 and Nup98 have similar intranuclear dynamics. (*A*) FRAP assays showing fluorescence recovery of GFP-Nup98 (panels *1–3*), sPom121-GFP (panels *4–6*), or GFP-Nup133 (panels *7–9*) during the time course shown at the *top*. The percent fluorescence recovery is indicated in the *bottom right* corner of each image, while the bleached area is indicated by the yellow dotted line. (*B*) Graph showing fluorescence recovery of either GFP-Nup98 (blue), sPom121-GFP (red), or GFP-Nup133 (green) under wild-type cell conditions. (*C*) FRAP assays showing fluorescence recovery of GFP-Nup98 (panels *1–3*), sPom121-GFP (panels *4–6*), or GFP-Nup133 (panels *7–9*) in the presence of the transcriptional inhibitor actinomycin D (Act. D) during the time course shown at the *top*. The percent fluorescence recovery is indicated in the *bottom right* corner of each image, while the bleached area is indicated by the yellow dotted line. (*D*) Graph showing fluorescence recovery of either GFP-Nup98 (blue), sPom121-GFP (red), or GFP-Nup133 (green) in cells treated with actinomycin D.

The observed colocalization of *sPom121* and *Nup98* and the previously defined *Pom121–Nup98* interaction (Mitchell et al. 2010) suggested that both proteins might function together in the nucleoplasm of human cells to carry out an unidentified function. As a first step to test whether the function of *sPom121* in the nucleoplasm is related to that of *Nup98*, we expressed either GFP-*Nup98* or *sPom121*-GFP in HeLa-C cells and conducted fluorescence resonance after photobleaching (FRAP) experiments. If both proteins are present in the same complex, we expected to observe similar recovery kinetics of intranuclear *sPom121* and *Nup98*. As shown in Figure 4A, after a short bleach, GFP-*Nup98* fluorescence recovery was identical to that of *sPom121*-GFP (cf. panels 2,3 and 5,6; also, Fig. 4B, cf. the blue trace and the red trace). We wondered whether all Nups that colocalize with *Nup98* share the same kinetics or whether *Nup98* and *sPom121* are different. To test this, we expressed GFP-tagged *Nup133*, a component of the *Nup107/160* complex that has also been shown to colocalize with *Nup98* in nucleoplasmic foci (Morchoisne-Bolhy et al. 2015). Unlike *Nup98* and *sPom121*, intranuclear GFP-*Nup133* fluorescence recovers slowly following a photobleach (Fig. 4A [panels 8,9], B [green trace]) suggesting that *sPom121* and *Nup98* might be in a subcomplex distinct from *Nup133* in the nucleoplasm. These results indicate that *sPom121* and *Nup98* might have a shared function in some intranuclear process.

Previous studies demonstrated that *Nup98* is a potent transcription factor in the nucleoplasm whose dynamics on chromatin are blocked by transcriptional inhibitors (Griffis et al. 2002, 2004; Capelson et al. 2010; Kalverda and Fornerod 2010; Pascual-Garcia et al. 2014). For example, when cells were treated with actinomycin D, the fluorescence recovery of *Nup98* by FRAP was much slower as *Nup98* becomes immobilized on chromatin and unable to recycle to intranuclear foci (Griffis et al. 2002, 2004). We hypothesized that *sPom121* cooperates with *Nup98* to regulate transcription at *Nup98* target genes and predicted that its dynamics would be similar to those of *Nup98*. To test this, we expressed either GFP-*Nup98* or *sPom121*-GFP in HeLa-C cells and conducted FRAP assays in the absence or presence of actinomycin D. Remarkably, the fluorescence recovery observed for *sPom121* matches the kinetics displayed by *Nup98* under all conditions (Fig. 4D). In contrast, GFP-*Nup133* displays different kinetics in the presence of actinomycin D (Fig. 4C,D). This suggests that *sPom121* and *Nup98* are likely part of the same complex, the dynamics of which are dependent on the transcription status of the cell.

### Nup98 and sPom121 cobind many promoters in human cells

**Figure 5.**
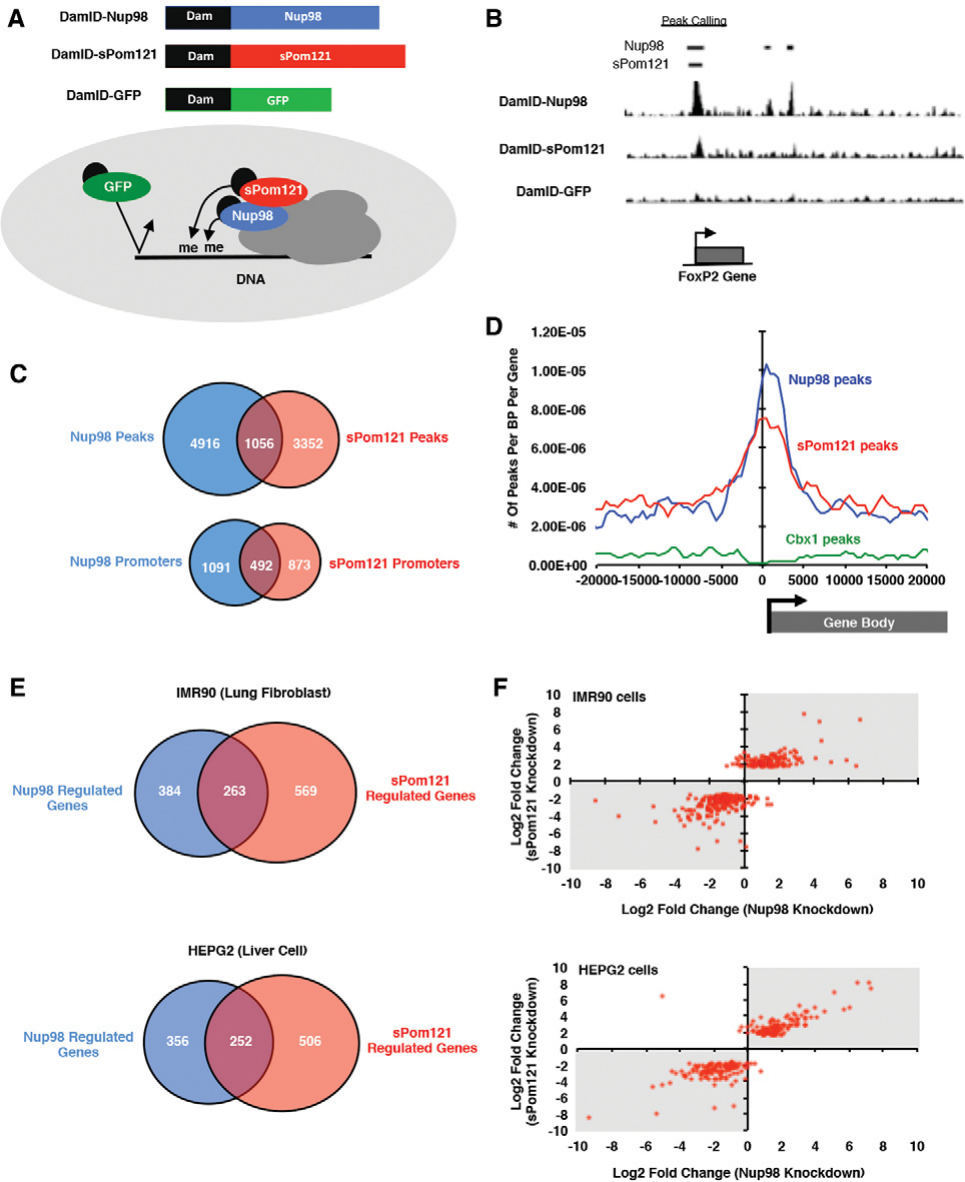
Nup98 and sPom121 cobind promoters in human cells. (*A*) Schematic of DamID constructs used to identify genomic binding sites of Nup98 (blue), sPom121 (red), and GFP (green). (*Bottom* diagram) DamID-tagged proteins that interact specifically with chromatin are expected to leave a well-defined peak, while GFP should not interact with chromatin and can be used to measure background DNA methylation. (*B*) Example of Nup98- and sPom121-binding peaks on a representative gene, FoxP2. Peaks that were called are shown at the *top* (*C*) Venn diagram showing overlap of Nup98 (blue) and sPom121 (red) DamID peaks (*top*) and Nup98 and sPom121 peaks that overlap at promoters (*bottom*) in HeLa-C cells. (*D*) Graph showing the number of peaks per base pair per gene relative to their transcriptional start sites. The *X*-axis represents location relative to the transcription start site (0 on the graph), while the *Y*-axis is the number of peaks per base pair per gene. Peaks identified in DamID experiments with Nup98 (blue), sPom121 (red), and a negative control protein, Cbx1 (green), which binds repressive chromatin are shown. (*E*) Overlap of genes misregulated by sPom121 (red) and Nup98 (blue) knockdowns. We asked what percentage of genes that are misregulated in sPom121 data sets (*P*-value < 0.01) is also misregulated by Nup98 knockdown (*P*-value < 0.01). The red area of the graph represents genes that were misregulated by sPom121 knockdown, while the blue represents genes that were misregulated by Nup98 knockdown. (*F*) Graph showing the log_2_ fold change of genes significantly misregulated (adjusted *P*-value < 0.05) in sPom121 knockdown data sets (*Y-*axis) plotted versus the log_2_ fold change of those same genes in Nup98 knockdown data sets (*X*-axis). If genes were up-regulated or down-regulated by both knockdowns, a red dot appears in the *top right* or *bottom left* quadrant of the graph, respectively (gray area of graph). In contrast, if a gene was misregulated by knockdown of either sPom121 or Nup98 but not by the other protein, a red dot appears in the *top left* or *bottom right* quadrant of the graph (white space).

*Nup98* was previously shown to bind to intranuclear gene promoters to regulate transcription (Capelson et al. 2010; Kalverda and Fornerod 2010; Liang et al. 2013; Light et al. 2013). This raised the interesting possibility that *sPom121*, via its loss of membrane anchoring and NPC targeting, might have acquired the ability to bind to *Nup98* target genes independently of localization at the nuclear periphery. This would allow it to expand its transcriptional role beyond that of the original *Pom121* protein. To address this, we attempted to determine the genome-wide binding pattern of *sPom121* using a DamID (DNA adenine methyltransferase [DAM] identification) approach (van Steensel and Henikoff 2000). We fused a DAM tag to *Nup98*, *sPom121*, or a control protein GFP (Fig. 5A) and expressed them in human HeLa-C cells. After extraction, methylated DNA was processed and subjected to deep sequencing and mapping against the reference human genome assembly. Peaks that were significantly above GFP-DamID background levels were counted as positive hits (Fig. 5B).

Intriguingly, we observed significant cobinding of *Nup98* and *sPom121* at 1056 genomic sites, 492 of which were co-occupied promoters (Fig. 5C). As compared with other genomic elements, *Nup98* and *sPom121* DamID results revealed a strong preference for binding in close proximity to the transcriptional start sites of genes (Fig. 5D), which is similar to what has been shown previously for *Nup98* and is indicative of a role in transcriptional initiation (Capelson et al. 2010; Kalverda and Fornerod 2010; Liang et al. 2013). When we performed DamID experiments with a DamID-*Nup98* mutant (DamID-*Nup98ΔCTD*) that has dramatically reduced affinity for the NPC (Griffis et al. 2002; Kalverda and Fornerod 2010), we found that its chromatin-binding profile is very similar to *sPom121* and wild-type *Nup98* (Supplemental Fig. S3C). Of the 1056 regions bound by both *Nup98* and *sPom121*, 580 of those peaks (55%) were also bound by intranuclear-specific *Nup98ΔCTD*, suggesting that the majority of *sPom121* targets are in the nucleoplasm.

To determine whether *sPom121* and *Nup98* affect the expression of their target genes, we depleted either *sPom121* or **Nup98** with siRNAs and observed the transcriptional consequences of this knockdown. For the former, we needed to specifically deplete *sPom121* protein without altering levels of *Pom121*, which would reduce NPC number and potentially perturb nuclear transport. To achieve this, we targeted the *sPom121* siRNA to exon 3 of the *sPom121* 5′ UTR that is unique from the *Pom121* mRNA (Supplemental Fig. S3D) and is not well conserved with the 5′ UTR of the *RB-associated KRAB zinc finger* (*RBAK*) gene from which the *sPom121* 5′ UTR originated. As shown in Supplemental Figure S3E, qPCR results in HeLa-C cells showed that, while *sPom121* RNA was efficiently depleted, as determined by the reduction of expression of the first 3 exons of *sPom121* (left graph, red bar), levels of the *Pom121* RNA remained stable, as measured by the levels of the TM-encoding exon 4 of *Pom121* (right graph, red bar).

Having an experimental system in place to study the role of *sPom121* in transcription without affecting nuclear transport or NPC density (Supplemental Fig. S4A,B), we focused on genes that were strongly bound by *sPom121* and *Nup98*. These included genes encoding transcription factors such as *Myc* and *TFAP2A* as well other targets involved in diverse cellular processes, such as cell signaling and Golgi apparatus function (*Dkk1* and *Ext1*) (Supplemental Fig. S3A). qPCR results showed that many of the genes tested, including *Myc*, *TFAP2A*, *Ext1*, and *Dkk1*, were up-regulated by both *sPom121* knockdown and **Nup98** knockdown (Supplemental Fig. S3B), suggesting an important role for *sPom121* in gene repression in HeLa-C cells. We also found a small number of target genes that were down-regulated in the presence of either the *sPom121* or *Nup98* siRNA, suggesting that *sPom121–Nup98*-dependent gene regulation can vary depending on the target. We therefore conclude that *sPom121* and *Nup98* cooperate to regulate the expression of target genes involved in diverse cellular processes in HeLa-C cells.

We next wondered whether *sPom121* plays a role as a transcriptional regulator in cells derived from different tissues. To test this, we used siRNAs to deplete *sPom121*, **Nup98**, or a control protein (*Fluc*) in two different cell types: human IMR90 lung fibroblasts and HEPG2 human liver cells. We conducted gene expression analysis using RNA-seq technology. We found that many genes were significantly misregulated in IMR90 (832 genes) and HEPG2 (758 genes) cells in the presence of *sPom121* siRNA (*P*-value < 0.01) (Fig. 5E). We observed similar results in cells treated with *Nup98* siRNA, where gene expression in IMR90 (647 genes misregulated) and HEPG2 (608 genes misregulated) cells was dramatically disrupted (Fig. 5E). Intriguingly, there is significant overlap between these two data sets; many of those genes that were misregulated in *sPom121* knockdown cells were also misregulated by *Nup98* knockdown in IMR90 (263 genes) and HEPG2 (252 genes) cell types (Fig. 5E, overlapping regions; Supplemental Figs. S5, S6). Importantly, genes were misregulated in the same direction when cells were treated with *sPom121* siRNA or *Nup98* siRNA (Fig. 5F, dots falling within the gray boxes indicate genes that changed in the same direction for both knockdowns). We conclude that *sPom121* cooperates with *Nup98* to regulate the expression of intranuclear genes in diverse cell types. Intriguingly, genes that are most misregulated in *sPom121* and *Nup98* knockdown cells are particularly important for homeostasis of the tissue in question (Supplemental Fig. S4C).

### Convergent evolution of sPom121-like homologs in mammals

**Figure 6.**
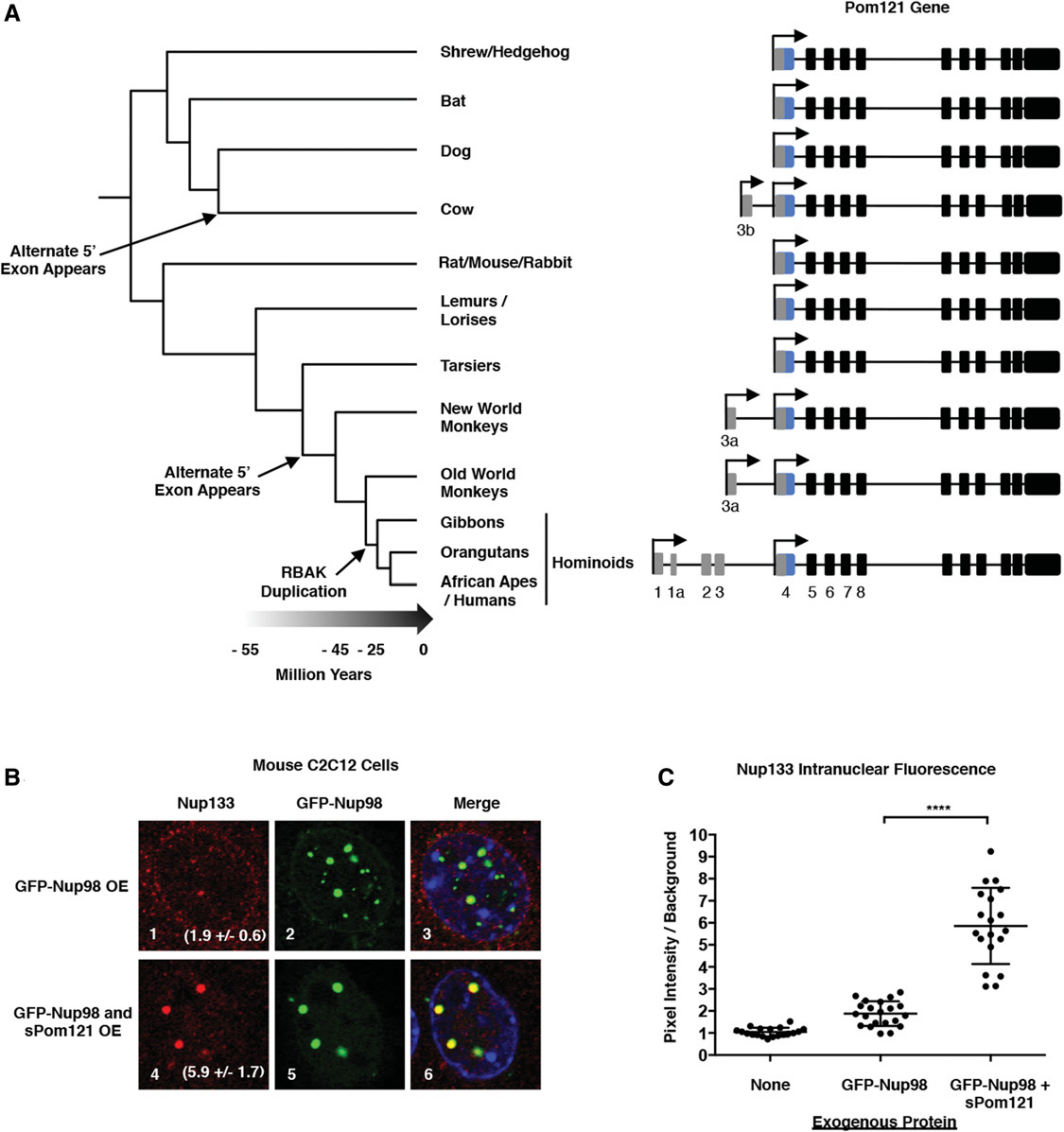
The hominoid version of sPom121 evolved recently and can recruit the Nup107/160 complex to the nucleoplasm when expressed in nonhominoid cells. (*A*) Novel upstream exons acquired sporadically during mammalian evolution are indicated in gray, while the Pom121 TM domain-coding exon (exon 4) is shown in blue, and the exons coding for the Pom121 ORF are shown in black. Note that, for simplicity, we are not showing the hominoid-specific duplication of the Pom121 gene (Pom121C). The gene structure of Pom121C is similar to the Pom121 locus, which is shown. (*B*) Localization of the endogenous Nup133 protein in mouse C2C12 cells in the presence of exogenous GFP-Nup98 or GFP-Nup98 and sPom121 together. The fold increase in Nup133 intensity in transfected cells relative to untransfected cells is shown in the *bottom right* corner of panels *1* and *4*. (*C*) Plot of the fluorescence intensity of intranuclear Nup133. The intensity of Nup133 intranuclear foci in transfected cells was measured and plotted relative to Nup133 intranuclear staining in untransfected cells (background).

We demonstrated that *sPom121* is produced in human cells via alternate transcriptional initiation that produces an mRNA with a 5′ UTR unique to the *Pom121* mRNA. We wondered whether the alternative transcription start site and unique 5′ UTR of *sPom121* are highly conserved or whether these features are recently evolved. We analyzed the conservation of the unique *sPom121* 5′ UTR in other metazoan species. Surprisingly, we found that the novel *Pom121* 5′ UTR arose when four exons from the *RBAK* gene, which encodes a retinoblastoma 1-binding protein, duplicated in a common ancestor of hominoids within the last 20 million–25 million years (Fig. 6A, bottom; gray exons 1–3). The *RBAK* gene duplication is positioned close to the 5′ end of the *Pom121* gene such that the *RBAK* transcriptional start site became an alternate start site for *Pom121* expression. The original *RBAK* ORF is not conserved at the *Pom121* locus, so these exons are now part of the 5′ UTR of *sPom121*. Additionally, as others have discussed previously (Kipersztok et al. 1995; Antonell et al. 2005; Funakoshi et al. 2007), we observed that the *POM121* gene locus was duplicated after the acquisition of the 5′ UTR of *RBAK*, resulting in expression of *Pom121* mRNAs from at least two different loci (*Pom121* and **Pom121*C*). The *Pom121* and **Pom121*C* loci appear to be functioning similarly to produce both *Pom121* and *sPom121* proteins, whereas an additional *Pom121* duplication, **Pom121*B*, does not appear to be active (Funakoshi et al. 2007). Although we focused the majority of our study on the *Pom121* locus, we designed our experiments to ensure that any observation or manipulation of *sPom121* or *Pom121* expression affected both the *Pom121* and **Pom121*C* loci.

The genomic rearrangement that brought the *RBAK* exons upstream of *POM121* appears to be exclusive to the ancestral hominoid lineage. However, we found that an alternative *Pom121* transcript lacking its TM domain has evolved on at least two other independent occasions during mammalian evolution. First, we identified ESTs from marmosets and rhesus macaques (New World and Old World monkey species, respectively) that correspond to alternative *Pom121* transcripts that use an alternative 5′UTR that lacks the TM-coding exon of *Pom121*, just like hominoid *sPom121* (Fig. 6A). However, in this case, only a single upstream exon is expressed (exon 3a), which again splices directly to the second canonical coding exon of *Pom121* (exon 5), bypassing the TM-coding exon (exon 4). Notably, the alternative *Pom121* exon in monkeys does not align with any sequence in hominoids. Together, these data suggest that the simian primate ancestor *Pom121* gene first acquired a novel alternative transcription start site and an upstream exon encoding a *sPom121* protein without a TM domain. Later, in the hominoid ancestor, the region containing that upstream exon was replaced by the *RBAK*-like exons, creating a different transcriptional isoform that nevertheless encodes the equivalent *sPom121* protein. In addition to primates, we found EST evidence for a third, independent evolutionary recruitment of alternative upstream exons encoding the *sPom121* protein in an ancestor of cows (Fig. 6A; “exon 3b”). Like in monkeys, the cow version of the alternative *Pom121* transcript expresses only one novel upstream exon, but the sequence is not homologous to any sequence found in monkeys or hominoids. Nevertheless, it results in a transcript predicted to encode a *sPom121*-like protein.

We next wanted to test whether the *sPom121* transcripts, which evolved independently in monkeys and cows, are also expressed like in humans. Using publicly available RNA-seq data from several different marmoset (New World monkey), rhesus macaque (Old World monkey), or cow tissue samples, we were able to observe expression of the noncanonical *sPom121* exon but only in the testes of marmosets and rhesus monkeys (Supplemental Fig. S7A,B). No *sPom121* expression was observed in the cerebellum, heart, kidney, or liver (Supplemental Fig. S7A,B). In cows, we found no evidence for expression of *sPom121* (brain, kidney, and liver tissues examined) (Supplemental Fig. S7C). These expression results strongly contrast with those in humans, in which we observed abundant *sPom121* mRNA in all tissues tested (Fig. 1D). This might reflect the better sampling of tissue types in humans relative to other primates or cows. Nevertheless, our findings highlight that alternate *sPom121*-like proteins have emerged via convergent evolution at least three times during mammalian evolution.

**Figure 7.**
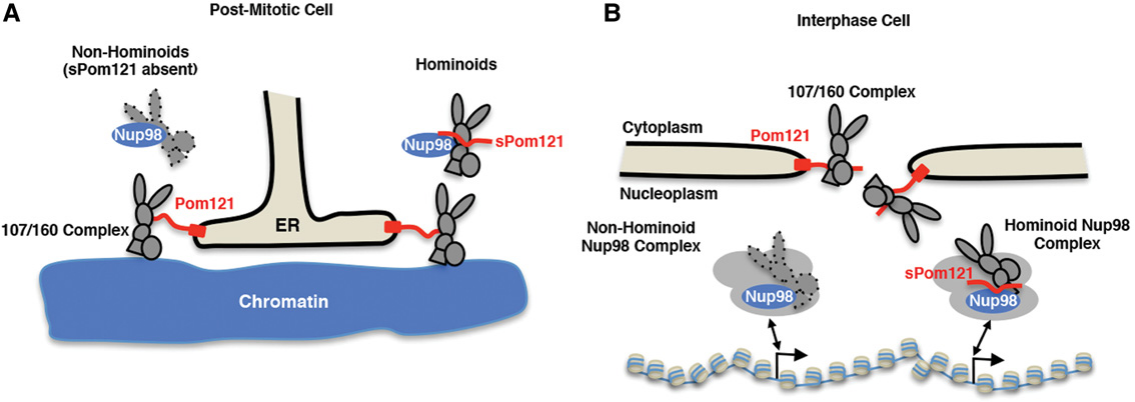
Model for sPom121-mediated recruitment of the Nup107/160 complex to the nucleoplasm of hominoid cells. (*A*,*B*, *left* side of model) In nonhominoid cells, sPom121 is likely not present, resulting in very little Nup133 (Nup107/160 complex) being prevented from entering the NPC during post-mitotic NPC assembly. (*A*,*B*, *right* side of model) In hominoids, sPom121 interacts with Nup98 and increases the affinity of Nup133 (Nup107/160) for Nup98 intranuclear complexes. As a result, a significant amount of Nup133 is retained in the nucleoplasm during post-mitotic and interphase NPC assembly. It remains to be seen whether the Nup107/160 complex can bind to Nup98 target genes and whether this has an effect on the regulation of said genes.

Interestingly, the widespread *sPom121* expression in hominoids seems to correlate with the appearance of nucleoplasmic *Nup107/160* complexes that are not associated with the NE (Morchoisne-Bolhy et al. 2015). Since a major role of *Pom121* is to recruit the *Nup107/160* complex to the NE during NPC assembly (Antonin et al. 2005; Doucet et al. 2010; Mitchell et al. 2010; Funakoshi et al. 2011; Talamas and Hetzer 2011), we predicted that *sPom121* (which lost its ability to integrate into the NE) might be able to redirect/retarget NPC scaffold proteins to non-NE locations in the nucleus. To test this idea, we expressed GFP-*Nup98* to induce the formation of *Nup98* foci that are not associated with the NE in mouse C2C12 cells that do not express *sPom121*. As expected for cells that do not express *sPom121*, we observed *Nup133* (a component of the *Nup107/160* complex) at NPCs but not strongly enriched at *Nup98* foci (Fig. 6B [panel 1], C). In contrast, ectopic expression of human *sPom121* resulted in the significant recruitment of *Nup133* to *Nup98* foci (Fig. 6B [panel 4], C). No *Nup133* intranuclear foci were observed in cells expressing *sPom121* alone (data not shown) possibly because intranuclear *Nup98* is limiting under these conditions. Our data suggest that *sPom121* along with Nup98 is sufficient to target the *Nup107/160* complex to non-NE sites. We conclude that in cells lacking *sPom121* (prior to hominoids), *Nup98* is not sufficient to recruit a significant amount of *Nup107/160* to the nucleoplasm (Fig. 7A [left side], B [left side]). However, in hominoid cells, *sPom121* is expressed and can compete with Pom121 for recruitment of *Nup107/160* complexes during the initial stages of mitotic and interphase NPC assembly (Fig. 7A [right side], B [right side]). Moreover, our results suggest that the recent appearance of *sPom121* might represent a crucial step in the functional evolution of not just *Pom121* but also the *Nup107/160* complex.

## Discussion

### sPom121 is a novel transcription cofactor for Nup98-mediated gene regulation

In this study, we provide evidence that a soluble form of *Pom121* exists in human cells that, together with *Nup98*, has the ability to bind to specific promoters to regulate their transcriptional output. Without its TM domain, the localization of *sPom121* and its functional properties become distinct from those of *Pom121*. Its nucleoplasmic localization allows *sPom121* to explore the entire genome regardless of nuclear membrane proximity. We show that *sPom121* colocalizes with *Nup98* and the *Nup107/160* complex via its NLS domain in an NPC-independent manner (Figs. 2B, 3B; Mitchell et al. 2010), thereby uncoupling transcription control from NPC function. *sPom121* and *Nup98* have nearly identical transcription-dependent dynamics in the nucleoplasm, suggesting that they mobilize in the nucleus in the same protein complex, which likely plays a direct role in transcription (Fig. 4A,B). Indeed, we found that *sPom121* and *Nup98* cobind to many gene promoters (Fig. 5C,D; Supplemental Fig. S3) in the nucleoplasm; knockdown of either protein misregulates an overlapping set of genes (Fig. 5E,F; Supplemental Figs. S5, S6). Although gene ontology results revealed some similarity among gene clusters that are misregulated across all cell types in the absence of *sPom121* and *Nup98*, our data suggest that *sPom121* regulates different gene sets depending on the cell type (Supplemental Figs. S4C, S5, S6). Overall, those genes most highly misregulated by *sPom121* depletion are usually also misregulated in the same direction by *Nup98* knockdown (Fig. 5E,F), offering further evidence that *sPom121* is cooperating with *Nup98* to regulate transcription.

### What is the role of sPom121 in mammalian cells?

What unique advantage is provided by the presence of *sPom121* that improves the ability of *Nup98* to regulate transcription in hominoids, monkeys, and cows? Recent studies in yeast have demonstrated the ability of the NPC to promote gene looping at the NPC, which allows rapid reinitiation of transcription after brief periods of repression (Casolari et al. 2004; O'Sullivan et al. 2004; Tan-Wong et al. 2009; Brickner and Brickner 2011). Furthermore, other studies have demonstrated that the NPC is an important scaffold at the nuclear periphery, where active genes and transcription factors congregate (Raices and D'Angelo 2012; Ptak et al. 2014; Ibarra and Hetzer 2015). The ability of the NPC to regulate gene expression is confined by its peripheral localization in yeast. Thus, it seems that evolution would favor a more mobile subset of Nups that can regulate gene expression anywhere in the nucleus, even beyond the NPC. Indeed, a subset of peripheral Nups like *Nup98* has acquired the ability to move off the NPC in metazoan species such as *Drosophila* (Capelson et al. 2010; Kalverda and Fornerod 2010). Interestingly, however, other than *Sec13*, no scaffold Nups have been shown to localize in the nucleoplasm in *Drosophila* cells, implying that gene expression regulation probably does not occur via an NPC-like scaffold in nonmammalian species but instead involves recruitment of chromatin-modifying complexes to chromatin by individual Nups (Capelson et al. 2010; Pascual-Garcia et al. 2014). The recent finding that the *Nup107/160* scaffold can exist away from the NPC in human cells raises the possibility that larger Nup subcomplexes probably exist in the nucleoplasm in mammalian cells (Morchoisne-Bolhy et al. 2015).

Our findings that even the TM Nup *Pom121* has evolved to be expressed in a soluble form provides further supporting evidence for the evolution of an intranuclear NPC subcomplex. Interestingly, *Pom121* protein was previously shown to interact with both *Nup98* and the *Nup107/160* complex, raising the possibility that *sPom121* promotes the interaction between *Nup98* and the *Nup107/160* complex in the nucleoplasm (Mitchell et al. 2010). Previous studies have demonstrated that *Pom121* promotes recruitment of the *Nup107/160* complex to chromatin as well as the interaction between nascent pores and reforming nuclear membranes during the early stages of post-mitotic NPC assembly (Fig. 7A,B). It is possible that *sPom121* competes with membrane-bound *Pom121* for interaction with the *Nup107/160* complex to promote prevention of a fraction of the *Nup107/160* complex from entering NPCs (Fig. 7A,B). Indeed, we demonstrated that, in contrast to human cells, *Nup107/160* complexes are not efficiently recruited to the nucleoplasm of mouse C2C12 cells. However, if exogenous *sPom121* is expressed, the *Nup107/160* complex (*Nup133*) is strongly recruited to *Nup98* complexes (Fig. 6B,C). These findings support the idea that nucleoplasmic localization of the *Nup107/160* complex simultaneously appeared with the evolution of *sPom121*. It will be interesting to investigate whether the *Nup107/160* complex can bind and play a role in the regulation of *Nup98*/*sPom121*-bound genes, which would provide further evidence for the assembly of an off-pore NPC scaffold functioning in gene regulation at intranuclear promoters.

### The Pom121 locus is rapidly evolving

One remarkable aspect of the *Pom121* gene is the hominoid-specific gene duplication and fusion with regulatory elements of the *RBAK* gene that resulted in the use of an alternative transcriptional start site (Fig. 6A). After *Pom121*'s appearance in vertebrates, it quickly gained an essential role in the regulation of interphase NPC assembly (Doucet et al. 2010; Dultz and Ellenberg 2010; Funakoshi et al. 2011; Talamas and Hetzer 2011; Field et al. 2014). The *Pom121* locus remained almost unchanged after this vertebrate-specific innovation (Antonell et al. 2005) until the divergence of mammals, where the expression of *sPom121* appears to have evolved at least three different times. What does this tell us about the importance of the *sPom121* protein? If convergent evolution is at play, it suggests that an important fitness benefit is provided by *sPom121* in not just human cells as we demonstrated but possibly other mammalian species. Unlike human cells, we did not see any expression of *sPom121* mRNA in monkeys outside of the testis or clear evidence that *sPom121* is expressed at significant levels in cows. Taken at face value, these expression studies suggest that the drastic genomic rearrangement of the *Pom121* gene in hominoids may have allowed for more widespread expression of *sPom121* than in other species. In the cow and monkey versions of *sPom121*, only one upstream exon is present, which arose from an unknown origin. In contrast, in hominoids, duplication of the *RBAK* gene resulted in the addition of three or four exons to the 5′ end of the *Pom121* gene and importantly donated a functional promoter (Fig. 1C), which might have been a breakthrough toward more ubiquitous *sPom121* expression.

Prior to hominoid divergence, the entire *Pom121* gene duplicated to form the *Pom121C* gene (Funakoshi et al. 2011). It is currently unclear whether the *Pom121* and *Pom121C* genes produce proteins with redundant functions. Interestingly, there are many amino acid changes as well as several insertions/deletions that have arisen since the duplication of *Pom121C*, which will give rise to a protein that differs considerably in amino acid sequence from that of the *sPom121* protein derived from the *Pom121* gene. In the future, it will be interesting to closely analyze the function of *sPom121* and *Pom121* proteins derived from the two *Pom121* loci to see whether they differentiated further in function. In turn, this might provide further insights into Nup-mediated transcriptional regulation and how it evolves.

In summary, we provide evolutionary and cell-biological evidence for the presence of a previously undetected soluble form of the TM Nup *Pom121*, which plays a dedicated role in transcription control. This transcription regulator contains all features of the NPC-associated *Pom121* except that it lacks the nuclear membrane-anchoring and NPC targeting domain. *sPom121* arose in the last common ancestor of hominoids from a rearrangement of genomic sequences that generated a new transcriptional start site, exhibiting a classic example of what Jacob (1977) called “molecular tinkering.” According to his idea, the appearance of a new molecular function is generated by alterations of pre-existing ones. In the context of the NPC, it appears that the adaptive advantage of generating *sPom121* as a soluble NLS-containing regulator appears to lie in the potential uncoupling of Nup-mediated gene regulation from the spatial constraints of NPC-mediated genome regulation at the nuclear periphery. This uncoupling allows the expansion of *Pom121*'s role in transcriptional regulation without compromising its role at the NPC, providing an elegant demonstration of the relief from the inherent antagonistic pleiotropy of encoding two important functions in the same Nup protein.

## Materials and methods

### IF and antibodies

HeLa-C cells (a gift from Volker Cordes and Maureen Powers) were cultured in DMEM/10% fetal bovine serum (FBS)/penicillin–streptomycin at 37°C in 5% CO_2_. For IF assays, cells were washed once with 1× phosphate-buffered saline (PBS) and fixed for 2 min in 4% paraformaldehyde (PFA). After three washes with PBS, cells were incubated for 10 min in 1× IF buffer (10 mg/mL BSA, 0.1% Triton X-100, 0.02% sodium dodecyl sulfate [SDS], 1× PBS). Next, cells were incubated for 2 h in IF buffer containing the antibodies of choice. The antibodies used included mouse Nup98 (1:100 dilution; Santa Cruz Biotechnology, C-5) (Fig. 2; Supplemental Fig. S2), rabbit Nup98 (1:500 dilution; Cell Signaling, P671) (Supplemental Fig. S2C, panels 2,17,23), Pom121 amino acids 236–552 (1:500 dilution [for IF] or 1:1000 dilution [for Western blot]; Genetex, GTX102128), Pom121 amino acids 1214–1229 (1:250 dilution; produced in the Hetzer laboratory), Pom121 amino acids 448–647 (1:500 dilution; produced in the Hetzer laboratory), rabbit Nup96 (1:500 dilution; NB100-93325), rabbit Nup133 against amino acids 1–22 (1:500 dilution; produced in the Hetzer laboratory), rabbit Nup107 (1:500 dilution; produced in the Hetzer laboratory), Elys (1:1000 dilution; produced in the Mattaj laboratory), Nup153 (1:1000 dilution; gift from B. Burke), TPR (1:500 dilution; Abcam, G00198), Nup50 (1:500 dilution; Abcam, G00318), Nup214 (1:1000 dilution; produced in the Hetzer laboratory), Nup358 (1:1000 dilution; Bethyl Laboratories, A301-796A), Nup88 (1:1000 dilution; BD Biosciences, 611896), Nup93 amino acids 2–218 (1:500 dilution; produced in the Hetzer laboratory), mAB414 (1:1000 dilution; Biolegend MMS-120R), and tubulin (1:1000 dilution [Western blot]; Sigma, T5168).

### Plasmids

All sPom121 constructs were cloned from a rat Pom121 construct that has been described previously (Doucet et al. 2010; Talamas and Hetzer 2011). Importantly, this rat Pom121 construct has been shown to function similarly to human Pom121 in all assays tested (Doucet et al. 2010; Talamas and Hetzer 2011). rPom121-3xGFP was made by amplifying rPom121 amino acids 1–1200 and ligating the N-terminal of a 3xGFP tag. sPom121-GFP and fragments thereof were made by amplifying the DNA fragments corresponding to the amino acids indicated in the figures (amino acid 1 of sPom121 corresponds to amino acid 241 of rat full-length Pom121) by PCR primers containing gateway-compatible sites. sPom121-Flag (Fig. 4B) was constructed by using a reverse oligo with a C-terminal Flag tag to amplify sPom121. Oligos also contained Gateway recombination sites that allowed for recombination into the pDonr207 vector. A subsequent recombination step was used to recombine sPom121-Flag into the pQXCIB retroviral vector. This vector was cotransfected with GFP-Nup98 (Fig. 4B). GFP-Nup98 was a gift from Jan Ellenberg. GFP-mNup133 has been described previously (Talamas and Hetzer 2011). PCR fragments were recombined into Donr207 and subsequently recombined into pDest47. The vector used for DamID was made by PCR-amplifying and ligating the heat-shock promoter, N-terminal Eco-Dam tag, V5-tag, and gateway recombination sites (RFC.1) from the pLgw-EcoDam-V5-RFC vector (a gift from B. van Steensel's laboratory) into the polylinker of the pMSCVpuro retroviral construct (pMSCVpuro-DAMID). cDNA corresponding to hNup98 amino acids 1–863, hNup98 amino acids 1–504 (DamID-Nup98ΔCTD), rPom121 amino acids 241–1200, or GFP were recombined into the pDonr207 vector and subsequently recombined into the RFC region of the pMSCVpuro-DAMID vector.

### Detergent extraction of sPom121

HeLa-C cells were grown in 15-cm plates with or without Pom121 siRNA. Twenty-five million cells (usually a confluent 15-cm dish of HeLa-C cells) per sample were used to extract sPom121 and subsequently Pom121. For sPom121 extraction, cells were washed with 1× PBS. Next, cells were placed in 10 mL of PBS and subsequently removed from the plates with a cell scraper. After spinning the cells in 10 mL of conicals for 5 min at 4°C, PBS was removed, and cells were incubated in 1 mL of mild extraction buffer (0.2% NP-40, 10 mM Tris 7.5, 150 mM NaCl, 1 mM EDTA, protease inhibitors) for 25 million cells for 2 min. Next, cells were spun at 3000 relative centrifugal force (rcf) for 3 min at 4°C to pellet insoluble material. Soluble nuclear lysates were removed and placed in a separate tube, of which 200 µL of lysate was placed with 200 µL of 2× SDS sample loading buffer and used as the soluble sample on Western blot. To extract membrane-bound components, including Pom121, insoluble pellets remaining after soluble protein extraction were incubated with 2 mL of harsh lysis buffer (0.2% NP-40, 0.25% sodium deoxycholate, 0.05% SDS, 10 mM Tris 7.5, 150 mM NaCl, 1 mm EDTA, protease inhibitors) for 2 min. Samples were spun at 10,000 rcf for 5 min, and 200 µL of supernatant was combined with 200 µL of 2× SDS load buffer. This sample was used as the membrane-bound fraction in Western blot.

### Detection of sPom121 by Western blot

Lysates obtained from mild detergent extraction were run on 4%–12% gradient gels at least until the point at which the protein marker corresponding to ∼75 kDa ran off the bottom of the gel. This allowed maximum separation between bands running at higher molecular weights (i.e., separating full-length Pom121 from sPom121). Following transfer of SDS gels to Western blots, cells were probed overnight at 4°C with Pom121 antibody (1:1000 dilution; Genetex, GTX102128). Western blots were developed using supersignal pico chemiluminescent substrate (Thermo Scientific). Alternatively, for a cleaner Western blot of sPom121, a coimmunoprecipitation of sPom121 could be conducted. After mild detergent extraction of sPom121 from 25 million cells, 5 µL of Pom121 antibody was added to soluble lysate and incubated 4 h with rotation at 4°C. Twenty-five microliters (bead volume) of protein A sepharose was added, and samples were incubated with rotation for >2 h at 4°C. Beads are then washed five times with mild lysis buffer and collected by centrifugation after each wash. After aspirating the last wash, 25 µL of 2× SDS load buffer was added. Beads were boiled for 3 min and loaded onto SDS-PAGE.

### 5′ RACE to clone Pom121 mRNAs

Two million cells of the specified cell type were pelleted by centrifugation and lysed with buffer RLT from the Qiagen RNAeasy kit. RNA was subsequently purified using the RNAeasy protocol. Five micrograms of RNA from each sample was used in a cDNA reaction to specifically amplify 5′ ends of Pom121 mRNAs using SuperScript II reverse transcriptase (Life Technologies). The oligo used for Pom121 reverse transcription anneals to a common region shared between sPom121 and Pom121. The oligo sequence was 5′-TTTCTCTTCCAGAGCTGTGAGATGCC-3′. Next, an anchor primer was ligated to the 3′ end of the Pom121 cDNA products (4 µL of Pom121 cDNA, 2 µL of phosphorylated anchor primer, 2 µL of RNA ligase buffer, 8 µL of 50% PEG 8000, 1 µL of 10 mM ATP, 1 µL of 0.1 M DTT, 1 µL of SS RNA ligase 1 [New England Biolabs]) overnight at 25°C. The anchor primer sequence was 5′-TTTAGTGAGGGTTAATAAGCGGCCGCGTCGTGACTGGGAGCGC-3′ amine. Next, a primer that anneals to Pom121 and a primer that anneals to the anchor primer were used to amplify Pom121 5′ cDNA fragments. Both primers contain 5′ gateway recognition sites for subsequent cloning into pDonr207. The sequences of these primers were Pom121 RACE reverse (5′-GGGGACCACTTTGTACAAGAAAGCTGGGTCCTCTGAGAATTGAGGCCTCTCTTCAG-3′) and anchor PCR forward (5′-GGGGACAAGTTTGTACAAAAAAGCAGGCTTCGCGGCCGCTTATTAACCCTCACTAAA-3′). After bacterial transformation, colonies were picked into LB and grown overnight at 37°C. DNA was purified using the Qiagen miniprep kit. Primers that anneal to the pDonr207 vector upstream of and downstream from the recombined product were used to sequence RACE clones. For evolutionary analysis (Fig. 4A), the 5′ UTR of sPom121 identified by RACE was blasted against other mammalian species to determine the first appearance of the RBAK exons in the locus.

### Knockdown of sPom121 and detection of sPom121 by qPCR

To obtain an efficient and specific knockdown of sPom121 mRNA, we designed an siRNA that targets the 5′ UTR that is unique to sPom121 and is not shared with Pom121 mRNA. Importantly, the sequence of this siRNA targets all sPom121 mRNAs regardless of whether they originate from the Pom121 or Pom121C locus. We also verified by RNA-seq that this siRNA does not target the mRNA of the Pom-ZP3 fusion. The target siRNA sequences used were sPom121 siRNA sense (5′-GCAACUUGCCCAAGUCCUUTT-3′) and sPom121 siRNA antisense (5′-AAGGACUUGGGCAAGUUGCTT-3′). Importantly, this siRNA targets a region of the sPom121 UTR that shares poor sequence homology with RBAK. To detect sPom121 by qPCR, we used a forward primer that anneals to the 5′ UTR sequence of sPom121 and a reverse primer that anneals to exon 5 of the Pom121 mRNA downstream from the TM-coding exon (see the Supplemental Material for sequences). For qPCR detection of sPom121 expression in 20 tissue types (Fig. 1D), we synthesized cDNA from RNA samples (Clontech, 636643) using SuperScript II reverse transcriptase (Life Technologies). Samples were subjected to qPCR using primers that specifically detect sPom121 or the TM domain of Pom121. Expression was normalized to actin.

### FRAP experiments

HeLa-C cells were transfected with sPom121-GFP (rPom121 amino acids 241–1200), GFP-Nup98 (a gift from Jan Ellenberg), or mNup133-GFP. After 48 h, cells were placed in an imaging chamber to simulate natural growth conditions (37°C and 5% CO_2_). A single focus was chosen and bleached to <30% initial fluorescence. Recovery of fluorescence was followed for 70 sec. For each time point, the average fluorescence from at least 20 cells was used to calculate a final measurement.

### DamID experiments

cDNA corresponding to sPom121, Nup98, Nup98ΔCTD, or GFP was cloned into MSCV-DamID-Gateway using the gateway system. Five micrograms of the MSCV-DamID vector of choice and 5 µg of Ampho were cotransfected into 293T cells. After 2 d, the medium was collected from the 293T cells and placed onto HeLa-C cells for 6 h in the presence of polybrene. After 48 h, cells were placed in medium containing 2 µg/mL puromycin. After 7–10 d, stable cell lines were obtained. DNA was harvested from 2 million cells using the Qiagen DNAeasy blood and tissue kit (catalog no. 69504). DNA (2.5 µg) was digested in a 20-µL reaction containing 1 µL of Dpn1 enzyme (New England Biolabs) and NEB buffer 4 at a 1× concentration for >4 h. The Dpn1-digested DNA was next ligated with the DamID adapter primer duplex. The sequences of the adapter duplex were AdR top (5′-CTAATACGACTCACTATAGGGCAGCGTGGTCGCGGCCGAGGA-3′) and AdR bottom (5′- TCCTCGGCCG-3′). To make the AdR duplex, 200 pmol of each primer were placed in an Eppendorf tube together, and the sample was diluted such that each primer was at a final concentration of 20 pmol/µL. The duplex was next heated for 3 min at 95°C and allowed to slowly cool for 15 min to room temperature. The resulting DNA duplex was placed in a ligation reaction with the Dpn1-digested DNA (20 µL of Dpn1-digested sample, 3 µL T4 ligase buffer [New England Biolabs], 3 µL of AdR duplex primers, 4 µL of ddH_2_O) for >4 h at 16°C. Five microliters of the ligation sample was amplified by PCR with KOD DNA polymerase (Toyobo) using the manufacturer's guidelines. The primer used to amplify the DNA fragments was bio-Adr-PCR (5′ bio-GGTCGCGGCCGAGGATC-3′). Note that the 5′ end of this primer contains a biotin label used in later purification steps. The resulting amplified DNA was diluted to 250 µL in binding and washing buffer (5 mM Tris-HCL at pH 7.5, 0.5 mM EDTA) and sonicated in Eppendorf tubes for 15 min using a Bioruptor sonicator (15 cycles of 30 sec on/30 sec off). The size of sonicated DNA was verified to be 200–500 bp before moving to the next step. Next, biotinylated ends were pulled down using Invitrogen Dynabeads MyOne Streptavidin T1. Fifty microliters was washed three times in binding and washing buffer containing 1 M NaCl. Next, the sonicated DNA was added to the 50 µL of washed Dynabeads. NaCl was added to the sample at this step to bring the concentration to 1 M. The DNA–bead mixture was rotated for 30 min at 4°C. The beads were then washed three times with binding and washing buffer + 1 M NaCl. The bound DNA was digested from the beads in 20 µL of DpnII digestion reaction for 2 h at 37°C. The sample was cleaned using a Qiagen MinElute Reaction CleaNup kit. The DNA was eluted in 20 µL, and libraries were prepared according to Illumina's specifications.

### RNA-seq analysis

The cell line indicated was transfected using RNAiMax (Invitrogen) and the desired siRNA at a final concentration of 25 nM. After 72 h, cells were washed in 1× PBS, and RNA was isolated using the RNeasy (Qiagen) purification kit. Libraries were prepared using the Illumina RNA library preparation kit. Reads were aligned to the human genome (hg19, GRCh37) using STAR (version 2.2.0.c) (Dobin et al. 2013). Only reads that aligned uniquely to a single genomic location were used for downstream analysis (mapping quality score [MAPQ] >10). Gene expression values were calculated for annotated RefSeq genes using HOMER by counting reads that overlapped exons (Heinz et al. 2010). Differentially expressed genes were found using EdgeR (Robinson et al. 2010). Genes that were significantly misregulated by sPom121 depletion (Fig. 5E [*P*-value < 0.01], F [adjusted *P*-value < 0.05]) were used for analysis. They were compared with genes that were misregulated by Nup98 knockdown (*P*-value < 0.01). Gene ontology functional enrichment analysis was performed using DAVID (Dennis et al. 2003).

Marmoset (GSE50747; WUGSC 3.2/calJac3), rhesus macaque (GSE30352; BGI CR_1.0/rheMac3), and cow (GSE43013; Baylor Btau_4.6.1/bosTau7) RNA-seq reads were aligned to their respective genomes. These data were obtained from previous studies (Brawand et al. 2011; Cortez et al. 2014; Fushan et al. 2015).

### ChIP-seq

Chromatin was fixed, and ChIP-seq was preformed as described previously (Liang et al. 2013; Jacinto et al. 2015). The antibody for H3K4me3 ChIP was purchased from Abcam (ab8580). Reads were aligned to the human genome (hg19, GRCh37) using bwa (version 0.7.12) (Li and Durbin 2009). Only reads that aligned uniquely to a single genomic location (MAPQ >10) were used for downstream analysis. ChIP-seq peaks and normalized bedGraph files were generated using HOMER using a false discovery rate (FDR) of 0.1% and fold enrichment over input of at least fourfold (Heinz et al. 2010).

### Identification of DamID-seq (DamID combined with next-generation sequencing) peaks

Reads were aligned to the human genome (hg19, GRCh37) using Bowtie2 (version 2.2.3) (Langmead and Salzberg 2012). Only reads that aligned uniquely to a single genomic location were used for downstream analysis. In addition, only reads aligning just downstream from a GATC sequence in the genome were kept for further analysis. Reads aligning to non-GATC locations were much more likely to represent nonspecific noise. Putative DamID-seq peaks were identified using HOMER in a manner similar to variable size ChIP-seq peaks, with several modifications as described previously (Jacinto et al. 2015). Duplicate reads, which are normally discarded to avoid artifacts in ChIP-seq, were retained for DamID-seq analysis, since most of the reads aligned to a limited number of GATC sites. DamID-seq peak regions were required to have twofold more normalized reads than GFP DamID-seq controls. In addition, peaks were required to contain at least 50 normalized reads per peak (per 10 million reads sequenced) to remove low-magnitude sites. Normalized bedGraph files were created for DamID by extending reads 1 kb both upstream and downstream to reflect the relative size of DamID-enriched regions. Annotation and comparisons between ChIP-seq peaks, DamID-seq peaks, and other genomic features such as the transcriptional start site were performed using the “mergePeaks” and “annotatePeaks.pl” programs in HOMER.

### Inducing Nup133 intranuclear localization in C2C12 cells

Mouse C2C12 cells were split onto coverslips at 30%–50% density 24 h prior to transfection. On the following day, cells were transfected with GFP-Nup98, sPom121-Flag, or both plasmids. After 24 h, cells were fixed in 2% PFA for 5 min. IF experiments were conducted as discussed above in the “IF and Antibodies” section. Nup133 intranuclear localization intensity was quantified using ImageJ software. GFP-Nup98 foci were circled in the green channel. Next, we switched to the red channel and recorded pixel intensity of Nup133 localization. The pixel intensity of Nup133 foci was compared with neighboring cells that did not overexpress GFP-Nup98 or sPom121-Flag. Untransfected cells did not display any obvious Nup133 intranuclear staining and thus were used as a background measurement. Plots of intranuclear fluorescence were constructed using Prism software.

## Supplementary Material

Supplemental Material
