# Supplementary material for: Evolution of a transcriptional regulator from a transmembrane nucleoporin
Source: Genes Dev. 2016 May 15;30(10):1155–71. doi: 10.1101/gad.280941.116 (PMC4888837; doi:10.1101/gad.280941.116)
Supplement: Supplemental Material [file supp_30_10_1155__index.html]

Evolution of a transcriptional regulator from a transmembrane nucleoporin — Evolution of a transcriptional regulator from a transmembrane nucleoporin — Supplemental Material 

# Evolution of a transcriptional regulator from a transmembrane nucleoporin

## Supplemental Material

- Supplemental\_Material.pdf
