# Supplementary material for: Evolution of a transcriptional regulator from a transmembrane nucleoporin
Source: Genes Dev. 2016 May 15;30(10):1155–71. doi: 10.1101/gad.280941.116 (PMC4888837; doi:10.1101/gad.280941.116)
Supplement: Supplemental Material [file supp_gad.280941.116_Supplemental_Material.pdf]

Supplemental Material

Oligonucleotide sequences used

sPom121 siRNA - sense - 5’ GCAACUUGCCCAAGUCCUUTT 3’

sPom121 siRNA - anti-sense – 5’ AAGGACUUGGGCAAGUUGCTT 3’

sPom121 qPCR – F - TAA CGG GAG GTG AAT TTC CA

sPom121 qPCR – R - ATG GAT CGG ATA GCG TCT TC

Pom121 TM domain – F - GAG CCC CGA GGT TCG CGC CCC

Pom121 TM domain – R - CAG GTA ACT GCC CAT GAG TAG

18s rRNA- F - GTG GAG CGA TTT GTC TGG TT

18s rRNA- R - CGC TGA GCC AGT CAG TGT AG

B-actin-qPCR1-F - GGA CTT CGA GCA AGA GAT GG

B-actin-qPCR1-R - AGC ACT GTG TTG GCG TAC AG

B-actin-qPCR2-F - GGC ATC CTC ACC CTG AAG TA

B-actin-qPCR2-R - GGG GTG TTG AAG GTC TCA AA

Ext1-F- ATT GTG ACA AGC CCC TAC CA

Ext1-R- TGA AGG CGA AAT CCA CCT CT

FoxP2-F- GCA ACT CTC ATA AGG CAG GC

FoxP2-R- TTT GTG ACC TTC GCT TCT GG

Myc-F- CTC CTG GCA AAA GGT CAG AG

Myc-R- TCG GTT GTT GCT GAT CTG TC

Dkk1-F- CCT TGG ATG GGT ATT CCA GA

Dkk1-R- TCC ATG AGA GCC TTT TCT CC

RBAK-F- TCG GAT GTC AAA CCT CAC TG

RBAK-R- TTG AAG GCT GAT CTG TGG TG

TFAP2A-F- GAT CCT CGC AGG GAC TAC AG

TFAP2A-R- TTG CTG TTG GAC TTG GAC AG

Supplemental Figure Legends

Supplemental Figure 1. Identification of a new Pom121 mRNA from which sPom121 is produced. (A) Agarose DNA gel showing migration of RACE clones from six different cell lines (B) An example of an sPom121 sequence identified by 5' RACE. The 5' adapter sequence is shown in orange, the 5' UTR of sPom121 is shown in black, the coding region of sPom121 is shown in red. (C) An example of Pom121 sequence identified by 5' RACE. The cloning adapter is shown in orange, while the 5' UTR is shown in black, exon 4 containing the TM domain of Pom121 is shown in blue and the coding region of Pom121 is shown in red. (D) Pom121 specific cDNA was made from HeLa-C, U2OS or IMR90 RNA. The 5' end of Pom121 mRNAs were cloned and sequenced. Clones containing the TM domain of Pom121 are shown in blue, while sPom121 specific exons are shown in grey. The region common to both Pom121 and sPom121 is shown in black. The number of clones that correspond to each transcript are indicated on the right. (E) Expression of other Nups relative to actin in various human

tissues and cell lines. (F) Correlation graphs of various Nups with sPom121 expression in various human tissues and cell lines. Relative sPom121 levels are graphed on the x-axis, while the comparative Nup of choice is mapped on the y-axis. The R^2 values and correlation coefficient (R) are shown in the upper left portion of the graph. In general expression of sPom121 correlated with expression of Pom121, Nup98 and especially Nup133. Only a moderate-weak correlation was observed between sPom121 expression and Nup93 or NDC1 expression.

Supplemental Figure 2. sPom121 and the Nup107/160 complex localize in the nucleoplasm in human cells (A) IF image showing co-localization of nucleoplasmic Pom121 (panel 1) with Nup98 (panel 2) using a third antibody that recognizes a different region than those antibodies used in Figure 1B. (B) IF assays showing localization of the Nup listed to the left of each row in HeLa-C cells (panels 1, 4, 7 and 10) as compared to Nup98 (panels 2, 5, 8 and 11). Merged images are shown in the right column (panels 3, 6, 9, and 12). The percentage of cells with the Nup co-localizing with Nup98 in the nucleoplasm is shown in the bottom right corner of the left panels, while the number of cells counted is shown in parenthesis. (C) IF assays showing localization of the Nup listed to the left of each row (panels 1, 4, 7, 10, 13, 16, 19 and 22) as compared to Nup98 (panels 2, 5, 8, 11, 14, 17, 20 and 23). Merged images are shown in the right column (panels 3, 6, 9, 12, 15, 18, 21, 24). The percentage of cells with the Nup co-localizing with Nup98 in the nucleoplasm is shown in the bottom right corner of the left panels, while the number of cells counted is shown in parenthesis. (D) IF assays showing localization of endogenous Pom121 (panels 1, 4 and 7) with GFP-Nup98 (panels 2, 5, and 8) in HeLa, U2OS or IMR90 cells. Merged images are shown in panels 3, 6, and 9. The percentage of cells with Pom121 co-localizing with GFP-Nup98 in the

nucleoplasm is shown in the bottom right corner of the left panels, while the number of cells counted is shown in parenthesis.

Supplemental Figure 3. sPom121 and Nup98 bind and regulate common genes in HeLa-C cells. (A) DamID traces from HeLa-C cells for the genes indicated at the bottom. Peaks are shown relative to a schematic of the gene. Peaks that were called by peak calling software are shown at the top. (B) qPCR expression results from HeLa-C cells treated with either Fluc (grey bar), sPom121 (red bar) or Nup98 siRNA (blue bar). (C) DamID traces from HeLa-C cells comparing sPom121 binding to a Nup98 mutant that cannot bind the NPC (Nup98ΔCTD). Peaks are shown relative to a schematic of the gene. Peaks that were called by peak calling software are shown at the top. (D) Schematic describing design of sPom121 siRNA. sPom121 siRNA is targeted to a sequence that is not shared with Pom121, RBAK or another gene that shares a similar 5'UTR to sPom121, PomZP3. (E) qPCR results showing expression of sPom121 and Pom121 in the presence of a control siRNA (Fluc) (blue bars) or the sPom121 siRNA (red bars).

Supplemental Figure 4. Global protein transport and NPC density are not affected by sPom121 knockdown. (A) Graph showing results of FRAP assays used to test fluorescence recovery of NLS-NES-tdTomato in cells treated with either Fluc siRNA (blue) or sPom121 siRNA (red). The nucleus of U2OS cells was bleached and the movement of cytoplasmic localized protein to the nucleus was observed for 180 seconds. (B) Measurement of NPC density in U2OS cells treated with siRNAs against Fluc, sPom121 or a positive control protein, Nup96. (C) Gene ontology of gene families

misregulated in sPom121 knockdown cells. The cluster, # of genes misregulated and p-values are indicated. The different cell types assayed are indicated on the left.

Supplemental Figure 5. sPom121 and Nup98 co-regulate genes in IMR90 cells. List of genes most misregulated when IMR90 cells are treated with an sPom121 or Nup98 siRNA. The gene name, Log2 fold change, p-value and adjusted p-value are indicated.

Supplemental Figure 6. sPom121 and Nup98 co-regulate genes in HEPG2 cells. List of genes most misregulated when HEPG2 cells are treated with an sPom121 or Nup98 siRNA. The gene name, Log2 fold change, p-value and adjusted p-value are indicated.

Supplemental Figure 7. sPom121 expression in marmoset, rhesus macaque and cow. (A-B) RNA-seq showing Pom121 expression profiles in various marmoset and rhesus macaque tissues. (C) RNA-seq showing expression profiles of Pom121 in cow tissues. The verticle axis is scaled for each track as not to crop the higher expressing peaks. Expression of Pom121 is approximately the same for all tissues except the testis where expression of Pom121 was typically 3-4 fold higher. Notably, no expression of the sPom121 specific exon (first exon) was observed in tissues besides testis even if the Y-axis was manipulated to enhance the size of the first exon (sPom121 specific exon). A red arrow is used to indicate where the RNA-seq peak corresponding to the sPom121 specific exon is expected to be located. This peak is visible in marmoset and macaque testis samples, but absent in cow tissues.

A

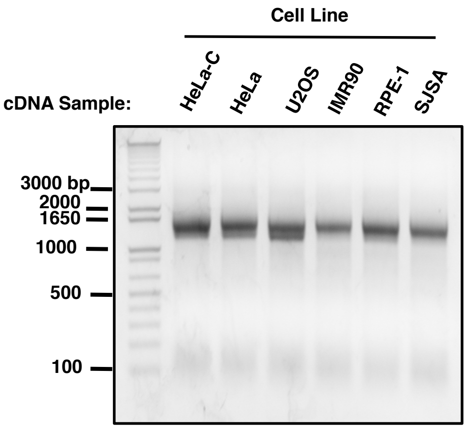

D

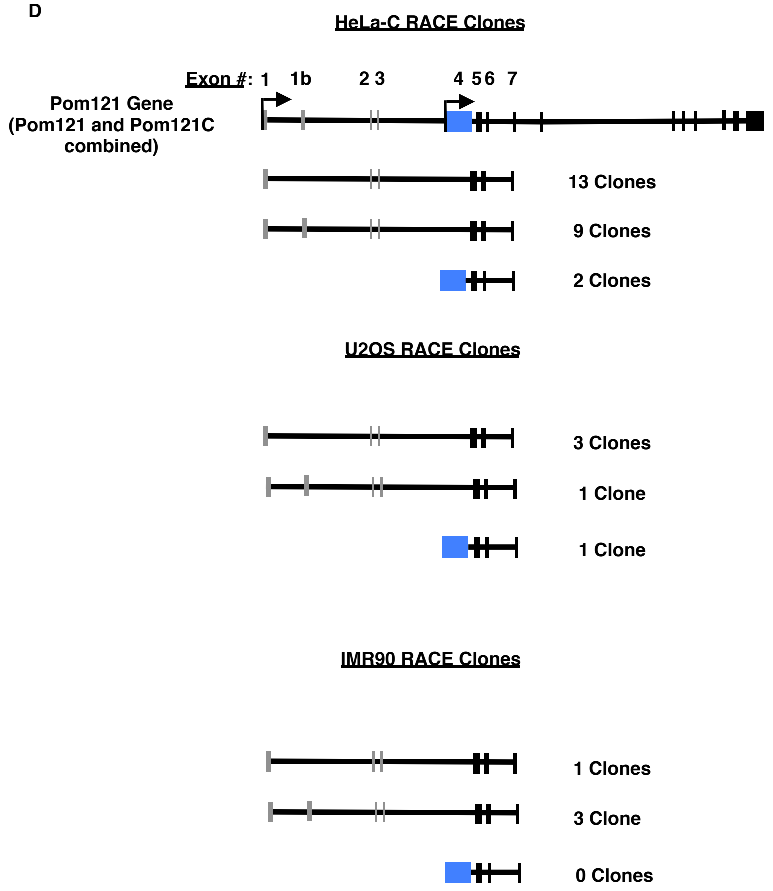

B

sPom121 RACE Clone

Cloning Adapter - GCGGCCGCTTATTAACCCTCACTAAAGGCCACCTCCGCACT
GCCCTCGCTTCCTGCGCCTCTTCAGGTCACCGCTTGCTCTA
GTTCCCAGGCTTTGGCCTCTAGTGGATGAGAATCACCGAGT
CTGCGGGGCTGGACGCTGACCGCCCGGGCCAGCACCTAGG
CGGGCGGGAGCTGTGCGGCCCAGGGTTCGCGCGGGCCGG
GTAGAGGCTCGAGCCGGGACCCCCGAGCGTGAACCCCGGA
GCCAGCGGCGCTGGGGCCAGAGGGGCCAGGCGGGAGGTG
GTGGCGGAGGCGAAGGGGCGACGGGACCTGGGCCTGGCC
5' UTR - CGTGTGTGTCCTCGGCGGCCTGGCGCCGGCCGTCGCTGTA
CGGTGAGCCCCAGGGAGGCGGATCTGGGCCCCGAGAAGG
ACACCCGCCTGGATTTGCCCCGTAGGCCCGGCCCGGGCCC
CTCGGGAGCAGAACAGCCTTGGTGAGGTGGACGGGAGGG
GACTTCGCGAGCAGACGCGCGCGCCAGCGACAGCAGCCC
GCCCCGGCCTCTCGGGAGCCGTGGGGCAGAGGCTGCAGA
GCCCCAGGAGGGGGCCAGTGTCATTCAAAGATGTGGCTGT
GGATTTCACCCAGGAGGAGTGGTGGCAACTGGACCCTGAT
GAGAAGATAACATACGGGGATGTGATGTTGGAGAACTACAG
CCATCTAGTTTCCCTGGCTTATGAGGTGGCAACATCTTGTAC
TTCGGAGATCTGAAGCCGAGCAACTTGCCCAAGTCCTTCTT
CTTTTCCCATTAACAAGATATGATATCACCAAGCCAAACGTC
ATCATTAAGTTGGAGCAGGGAGAGGAGCTGTGGATAACGGG
AGGTGAATTTCCATGTCAACATAGTCCAGGGATTGTGGGACT
TTACCAAATCGGTTTGTAATAACACCTAGAAGACGCTATCCG
ATCCATCAGGCCCAGTATTCCTGTCTGGGGGTACTTCCCACC
GTGTGCTGGAATGGTTATCACAAGAAGGCTGTGCTGTCCCC
TCGCAACTCCAGGATGGTGTGTAGCCCAGTGACTGTGAGGA
TCGCCCCTCCTGACAGAAGATTTTCGCGTTCTGCGATACCA
GAGCAGATAATCAGCTCAACACTGTCCTCACCATCAAGTAA
ORF sPom121 - CGCCCCAGACCCATGTGCAAAGGAGACAGTACTGAGTGCC
CTCAAAGAGAAGGAGAAGAAAAGGACAGTGGAGGAAGAA
GACCAAATATTCCTTGATGGCCAGGAAAATAAAAGAAGGCG
CCATGATAGCAGTGGCAGTGGACATTCAGCATTTGAGCCCC
TGGTGGCCAATGGAGTCCCCGCTTCTTTTGTGCCTAAGCCT
G

C

Pom121 RACE Clone

Cloning Adapter - GCGGCCGCTTATTAACCCTCACTAAAAAGTCTCCTCCGCG
5' UTR - GCGCGGAGCCGCGATGTCTCCGGCGGCTGCGGCGGCTG
GAGCAGGCGAGCGGCGGCGGCCGATAGCGAGTGTCAGG
GACGGCCGGGGCCGGGGCTGCGGCGGGCCGGCCGGGG
CGGCGCTTCTCGGCCTGTCGCTGGTCGGCCTCCTACTGT
ACCTCGTGCCTGCTGCGGCTGCGCTGGCCTGGCTGGCC
GTGGGGACTACCGCGGCCTGGTGGGGACTGAGCCGCGA
GCCCCGAGGTTCGCGCCCCTTGTCCTCCTTCGTTCAGAA
GGCGCGACATCGGCGAACACTGTTCGCTTCGCCTCCGGC
Exon4 (TM domain) - CAAGTCGACAGCCAACGGAAACCTCCTAGAGCCGCGGA
CCCTGCTCGAAGGACCTGACCCTGCCGAACTGCTCCTCA
TGGGCAGTTACCTGGGCAAGCCCGGGCCGCCGCAGCCC
GCCCCCGCTCCGGAGGGCCAGGACCTGCGGAATAGGCC
TGGCCGCCGCCCACCCGCCCGCCCGGCGCCGCGCTCCA
CACCGCCCTCCCCGCCGACCCATCGCGTTCACCACTTTT
ACCCCTCTCTCCCCACTCCTCTTCTCCGACCCTCCGGGA
GGCCTTCCCCACGGGATCGTGGGACTTTACCAGATCGGT
TTGTAATAACACCTCGAAGACGCTATCCGGATCCATCAGA
CCCAGTATTCCTGTCCGGGGGTACTTCCCACAGTGTGCT
GGAATGGTTATCACAAGAAGGCTGTGCTGTCCCCTCGCA
ACTCCAGGATGGTGTGTAGCCCAGTGACTGTGAGGATCC
ORF Pom121 - GCCCCTCCTGACAGAAGATTTTCACGTTCTGCGATACCAG
AGAGATAATCAGCTCACACTGTCGTCACATCAAGTATTGC
CCCAGACCCATGTGCAAAGGAGACTGTACTGAGTGCCCT
CAAAGAGAAGAAGAAGAAAAGGACAGTGGAGGAAGAA
GACCAAATATTCCTTGATGGCCAGGAAAATAAAAGAAGG
CGCCATGATAGCAGTGGCAGTGGACATTCAGCATTTGAG
CCCCTGGTGGCCAGTGGAGTCCCCGCTTCTTTTGTGCCT
AAGCCTGGGTCTCTGAAGAGAGGCCTCAATTCTCAGAGG
ACCCAGCTTTCTTGTACAAAGTTGGCATTATAAGAAAGCA
TTGCTTATCAATTTGTTGCAACGAACAGGTCACTATCAGT
CAAAATAAAATCATTATTGCCTCCAGCTGCAGTCGCCCC

E

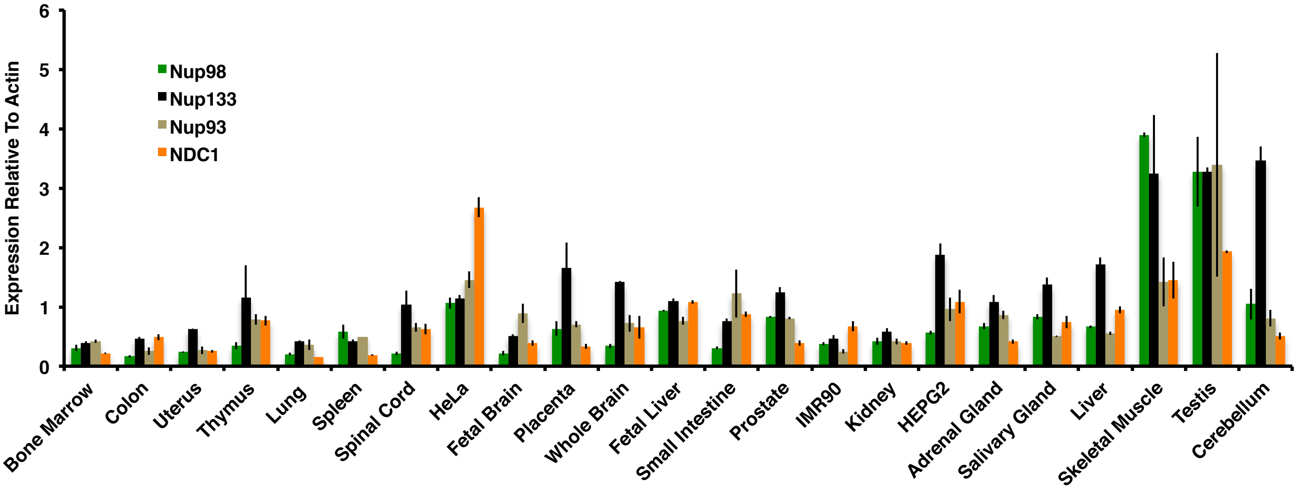
Nup98
Nup133
Nup93
NDC1
Expression Relative To Actin
Bone Marrow
Colon
Uterus
Thymus
Lung
Spleen
Spinal Cord
HeLa
Fetal Brain
Placenta
Whole Brain
Fetal Liver
Small Intestine
Prostate
IMR90
Kidney
HEPG2
Adrenal Gland
Salivary Gland
Liver
Skeletal Muscle
Testis
Cerebellum

F

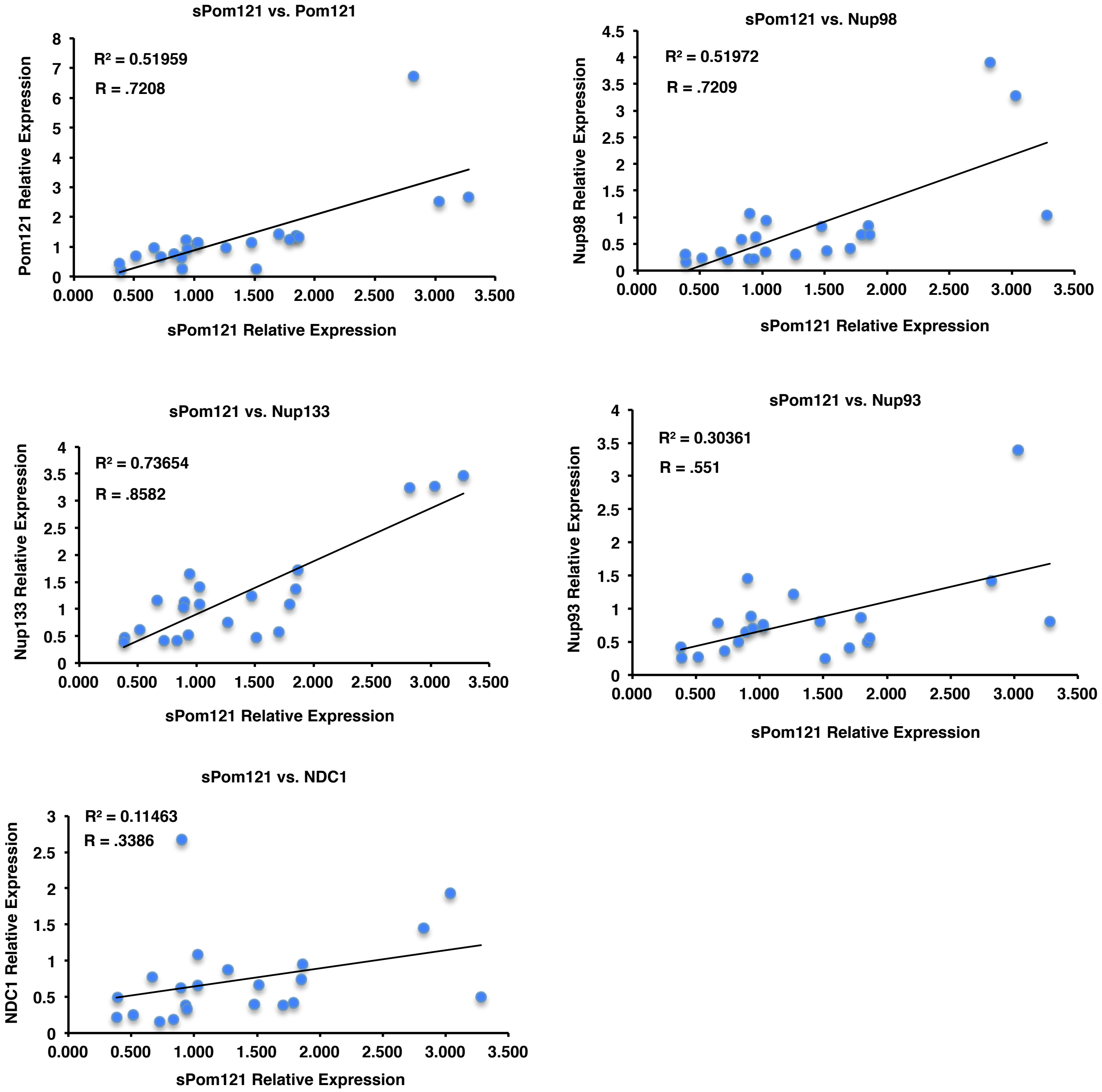
sPom121 vs. Pom121
R² = 0.51959
R = .7208
Pom121 Relative Expression
sPom121 Relative Expression
sPom121 vs. Nup98
R² = 0.51972
R = .7209
Nup98 Relative Expression
sPom121 Relative Expression
sPom121 vs. Nup133
R² = 0.73654
R = .8582
Nup133 Relative Expression
sPom121 Relative Expression
sPom121 vs. Nup93
R² = 0.30361
R = .551
Nup93 Relative Expression
sPom121 Relative Expression
sPom121 vs. NDC1
R² = 0.11463
R = .3386
NDC1 Relative Expression
sPom121 Relative Expression

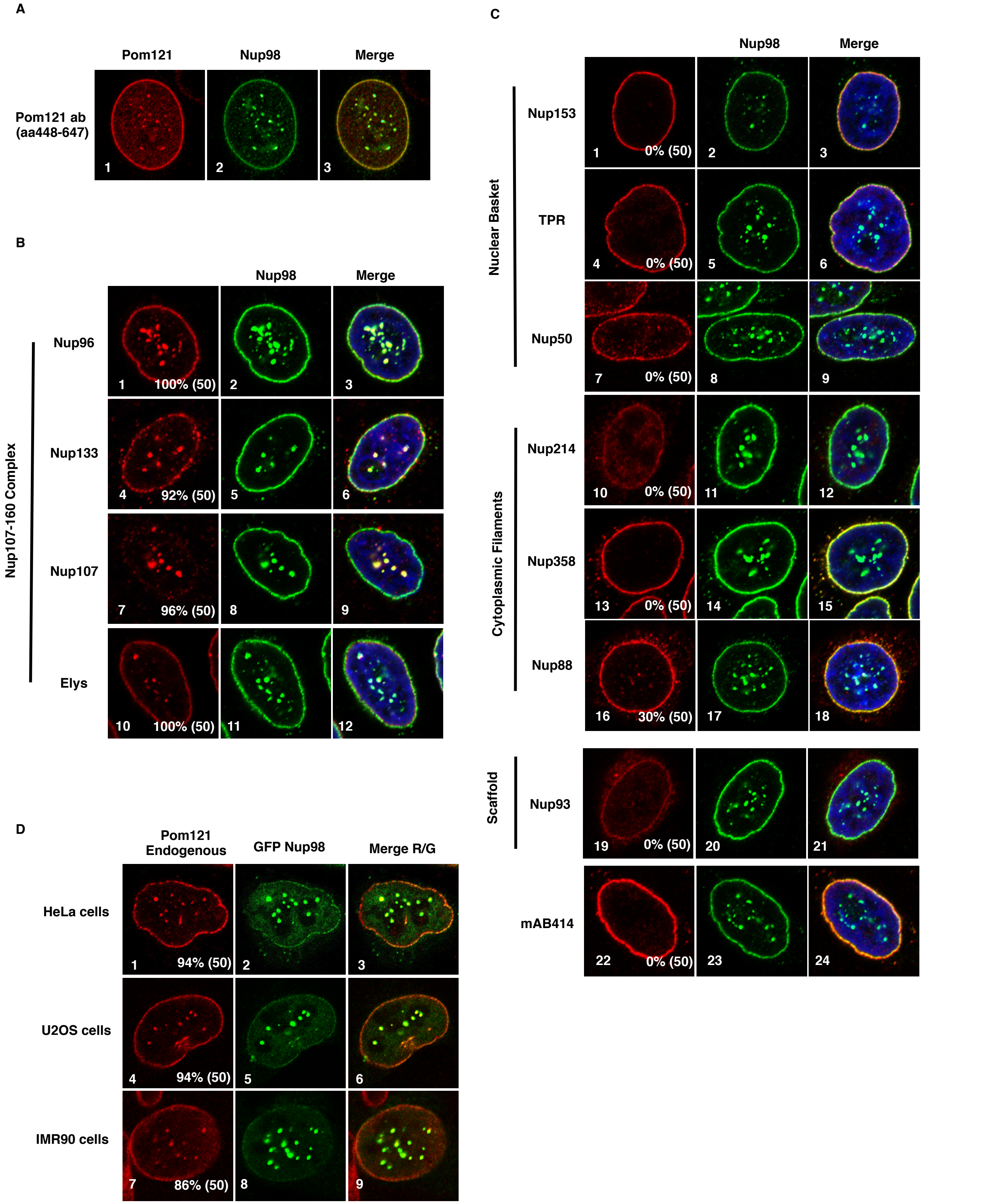
A
Pom121
Nup98
Merge
Pom121 ab (aa448-647)
1
2
3
B
Nup98
Merge
Nup107-160 Complex
Nup96
1
100% (50)
2
3
Nup133
4
92% (50)
5
6
Nup107
7
96% (50)
8
9
Elys
10
100% (50)
11
12
C
Nup98
Merge
Nuclear Basket
Nup153
1
0% (50)
2
3
TPR
4
0% (50)
5
6
Nup50
7
0% (50)
8
9
Cytoplasmic Filaments
Nup214
10
0% (50)
11
12
Nup358
13
0% (50)
14
15
Nup88
16
30% (50)
17
18
Scaffold
Nup93
19
0% (50)
20
21
mAB414
22
0% (50)
23
24
D
Pom121 Endogenous
GFP Nup98
Merge R/G
HeLa cells
1
94% (50)
2
3
U2OS cells
4
94% (50)
5
6
IMR90 cells
7
86% (50)
8
9

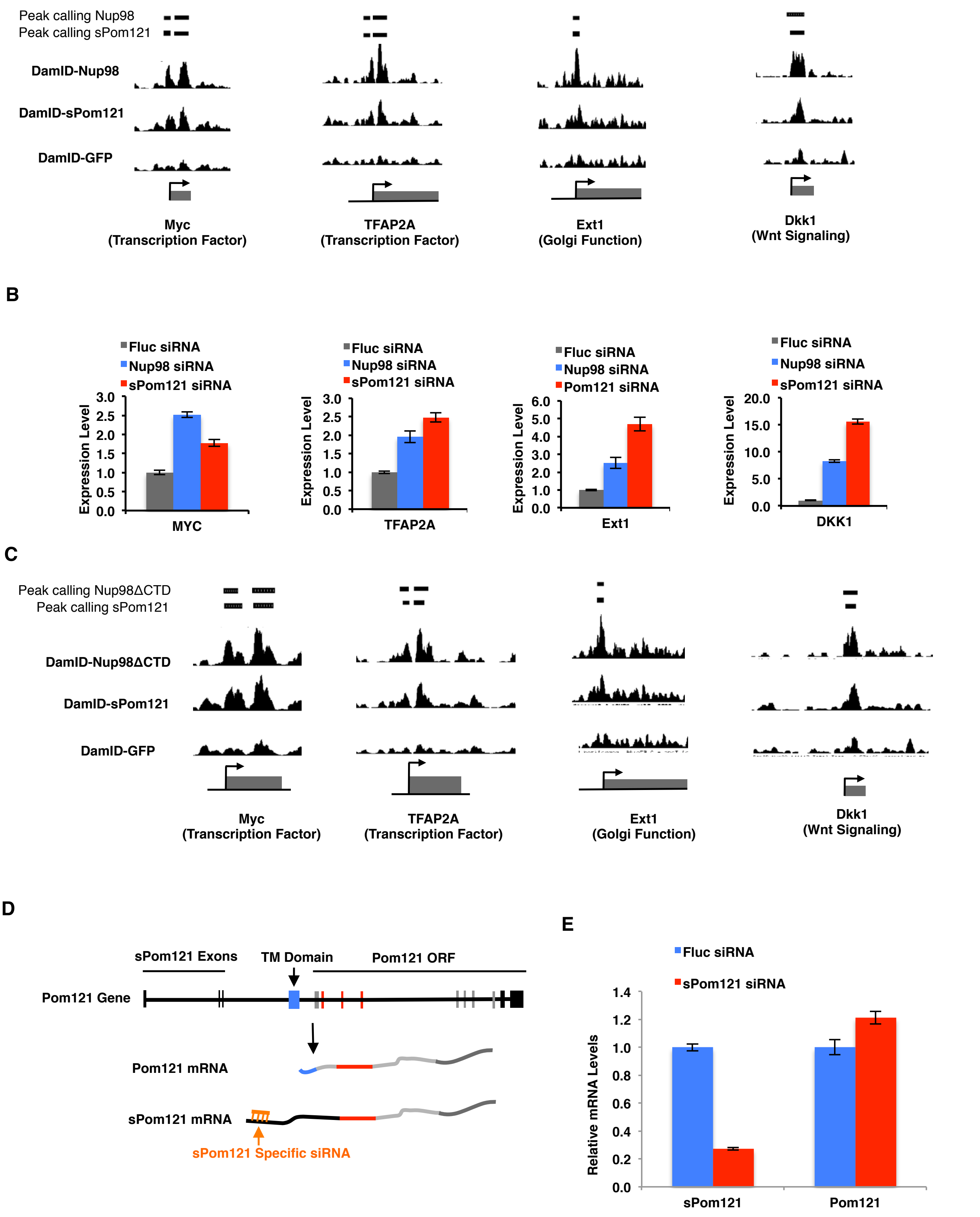
A
Peak calling Nup98
Peak calling sPom121
DamID-Nup98
DamID-sPom121
DamID-GFP
Myc
(Transcription Factor)
TFAP2A
(Transcription Factor)
Ext1
(Golgi Function)
Dkk1
(Wnt Signaling)
B
Fluc siRNA
Nup98 siRNA
sPom121 siRNA
Expression Level
3.0
2.5
2.0
1.5
1.0
0.5
0.0
MYC
Fluc siRNA
Nup98 siRNA
sPom121 siRNA
Expression Level
3.0
2.5
2.0
1.5
1.0
0.5
0.0
TFAP2A
Fluc siRNA
Nup98 siRNA
Pom121 siRNA
Expression Level
6.0
5.0
4.0
3.0
2.0
1.0
0.0
Ext1
Fluc siRNA
Nup98 siRNA
sPom121 siRNA
Expression Level
20.0
15.0
10.0
5.0
0.0
DKK1
C
Peak calling Nup98ΔCTD
Peak calling sPom121
DamID-Nup98ΔCTD
DamID-sPom121
DamID-GFP
Myc
(Transcription Factor)
TFAP2A
(Transcription Factor)
Ext1
(Golgi Function)
Dkk1
(Wnt Signaling)
D
sPom121 Exons
TM Domain
Pom121 ORF
Pom121 Gene
Pom121 mRNA
sPom121 mRNA
sPom121 Specific siRNA
E
Fluc siRNA
sPom121 siRNA
Relative mRNA Levels
1.4
1.2
1.0
0.8
0.6
0.4
0.2
0.0
sPom121
Pom121

A

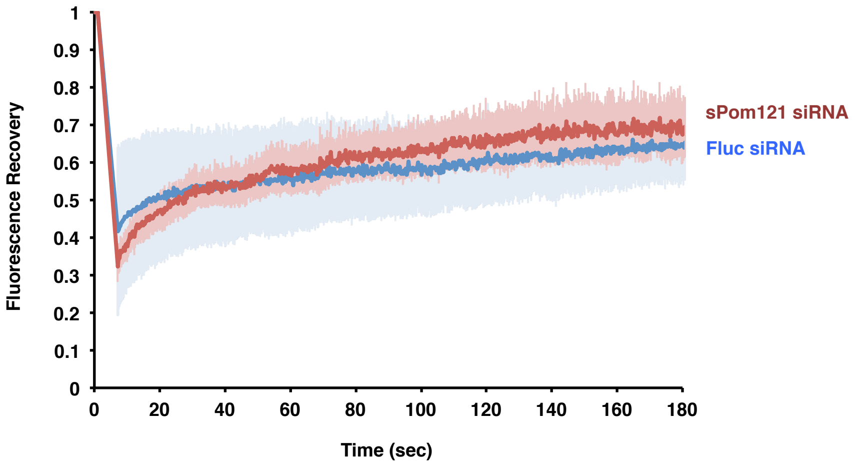
Fluorescence Recovery
1
0.9
0.8
0.7
0.6
0.5
0.4
0.3
0.2
0.1
0
0 20 40 60 80 100 120 140 160 180
Time (sec)
sPom121 siRNA
Fluc siRNA

B

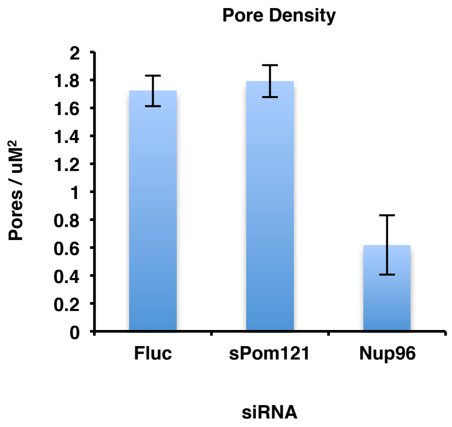
Pore Density
Pores / uM²
2
1.8
1.6
1.4
1.2
1
0.8
0.6
0.4
0.2
0
Fluc
sPom121
Nup96
siRNA

C

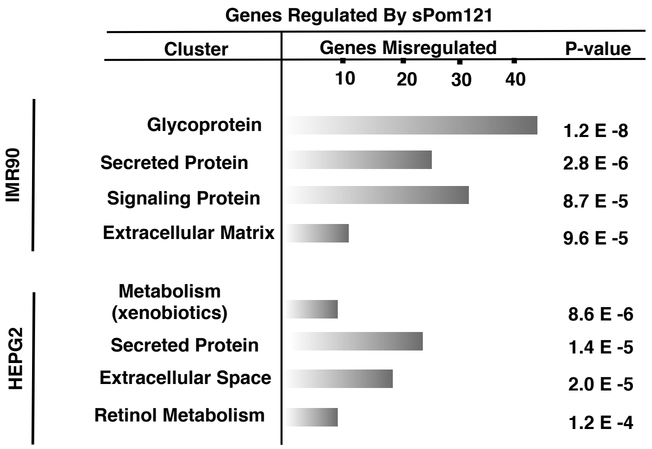
Genes Regulated By sPom121
Cluster
Genes Misregulated
P-value
10 20 30 40
IMR90
Glycoprotein
1.2 E -8
Secreted Protein
2.8 E -6
Signaling Protein
8.7 E -5
Extracellular Matrix
9.6 E -5
HEPG2
Metabolism (xenobiotics)
8.6 E -6
Secreted Protein
1.4 E -5
Extracellular Space
2.0 E -5
Retinol Metabolism
1.2 E -4

IMR90 Cells

MS# GENESDEV/2016/280941_Franks et al._Supp. Figure 5

Gene Name	Nup98 siRNA Log2 Fold Change	Nup98 siRNA P-Value	Nup98 siRNA Adjusted P-Value	sPom121 siRNA Log2 Fold Change	sPom121 siRNA P-Value	sPom121 siRNA Adjusted P-Value
PARM1ICipar1IDKFZP564O0823IPARM-1IWSC4I-I4q13.3-q21.3Iprotein-coding	-5.15	1.63E-19	7.12E-16	-4.70	1.75E-17	4.08E-13
CLDN1ICLD1IILVASCISEMP1I-I3q28-q29Iprotein-coding	4.40	4.27E-16	1.25E-12	4.61	3.11E-17	4.08E-13
SLITRK6IDFNMYPI-I13q31.1Iprotein-coding	-2.68	5.18E-08	2.16E-05	-4.29	6.44E-16	5.64E-12
TYRP1ICAS2ICATBIGP75IOCA3ITRPITRP1ITYRPIb-PROTEINI-I9p23Iprotein-coding	-3.29	2.06E-09	1.39E-06	-4.24	4.27E-13	2.80E-09
**CFTRIABC35IABCC7ICFICFTR/MRPIMRP7ITNR-CFTRIdJ760C5.1I-I7q31.2Iprotein-coding**	**-3.01**	**6.59E-09**	**4.03E-06**	**-3.90**	**6.37E-13**	**3.34E-09**
TMEM100I-I-I17q22Iprotein-coding	-1.92	1.26E-04	1.80E-02	-4.01	1.47E-12	6.42E-09
BMP5I-I-I6p12.1Iprotein-coding	-0.90	5.60E-02	1.00E+00	-3.71	2.35E-12	8.81E-09
NKD2INaked2I-I5p15.3Iprotein-coding	-1.21	2.05E-02	6.31E-01	-4.84	5.68E-12	1.86E-08
ABCA8I-I-I17q24Iprotein-coding	-2.54	1.67E-06	5.08E-04	-3.95	8.81E-12	2.57E-08
KCTD12IC13orf2IPFET1IPFETINI-I13q22.3Iprotein-coding	-2.10	1.16E-05	2.66E-03	-3.33	4.17E-11	1.09E-07
PDE1AICAM-PDE-1AIHCAM-1IHCAM1IHSPDE1AI-I2q32.1Iprotein-coding	-3.72	1.71E-08	8.01E-06	-4.40	1.24E-10	2.71E-07
IGF1IIGF-IIIGF1AIIGFII-I12q23.2Iprotein-coding	-2.18	5.59E-05	9.64E-03	-4.03	1.23E-10	2.71E-07
KCNJ2IATFB9IHHBIRK1IHHIRK1IIRK1IKIR2.1ILQT7ISQT3I-I17q24.3Iprotein-coding	-0.43	3.56E-01	1.00E+00	-3.39	1.75E-10	3.53E-07
HES1IHES-1IHHLIHRYIbHLHb39I-I3q28-q29Iprotein-coding	2.89	2.18E-07	8.18E-05	3.51	3.23E-10	6.05E-07
BRINP3IDBCCR1LIDBCCR1L1IFAM5CI-I1q31.1Iprotein-coding	-3.17	1.16E-06	3.80E-04	-4.59	5.51E-10	9.63E-07
CYP26B1ICYP26A2IP450RAI-2IP450RAI2IRHFCAI-I2p13.2Iprotein-coding	-1.55	3.27E-03	1.94E-01	-3.81	1.31E-09	2.16E-06
GUCY1B3IGC-S-beta-1IGC-SB3IGUC1B3IGUCB3IGUCSB3IGUCY1B1I-I4q31.3-q33Iprotein-coding	-2.89	9.23E-08	3.72E-05	-3.33	1.49E-09	2.18E-06
COL5A3I-I-I19p13.2Iprotein-coding	1.66	4.81E-04	5.07E-02	3.01	1.46E-09	2.18E-06
RGS18IRGS13I-I1q31.2Iprotein-coding	-0.42	3.96E-01	1.00E+00	-3.50	1.71E-09	2.36E-06
TNXBIEDS3IHXBLITENXITN-XITNXITNXB1ITNXB2ITNXBSIVUR8IXBIXBSI-I6p21.3Iprotein-coding	-3.15	3.01E-08	1.34E-05	-3.36	3.37E-09	4.42E-06
C16orf89I-I-I16p13.3Iprotein-coding	-2.67	8.08E-06	1.96E-03	-3.84	3.63E-09	4.54E-06
SPON1IVSGP/F-spondinIf-spondinI-I11p15.2Iprotein-coding	-3.37	9.90E-09	5.53E-06	-3.41	4.06E-09	4.84E-06
KCNK3IK2p3.1IOAT1IPPH4ITASKITASK-1ITBAK1I-I2p23Iprotein-coding	-3.31	8.56E-07	2.92E-04	-4.06	1.63E-08	1.86E-05
TSPAN12IEVR5INET-2INET2ITM4SF12I-I7q31.31Iprotein-coding	-1.78	1.69E-04	2.31E-02	-2.77	1.71E-08	1.88E-05
SERPINE1IPAIIPAI-1IPAI1IPLANH1I-I7q22.1Iprotein-coding	1.03	2.61E-02	6.99E-01	2.74	2.27E-08	2.38E-05
KCNG1IK13IKCNGIKV6.1IkH2I-I20q13Iprotein-coding	1.78	3.49E-04	3.97E-02	2.82	2.67E-08	2.69E-05
HRIALUNCIAUIHSA277165IHYPT4IMUHHIMUHH1I-I8p21.2Iprotein-coding	-1.53	1.70E-03	1.25E-01	-2.87	3.14E-08	3.01E-05
HTR2BI5-HT(2B)I5-HT2BI-I2q36.3-q37.1Iprotein-coding	-1.43	4.96E-03	2.56E-01	-3.08	3.31E-08	3.01E-05
ITGB8I-I-I7p21.1Iprotein-coding	-0.42	3.63E-01	1.00E+00	-2.76	3.33E-08	3.01E-05
ST8SIA1IGD3SISIAT8ISIAT8-AISIAT8AIST8SiaII-I12p12.1-p11.2Iprotein-coding	-2.05	3.55E-03	2.04E-01	-5.35	4.21E-08	3.68E-05
KCNQ5IKv7.5I-I6q14Iprotein-coding	0.25	6.83E-01	1.00E+00	2.88	4.42E-08	3.74E-05
OLFM2INOE2INOELIN2INOELIN2_V1IOlfCI-I19p13.2Iprotein-coding	1.26	8.65E-03	3.73E-01	2.71	4.63E-08	3.80E-05
SULF1IHSULF-1ISULF-1I-I8q13.2Iprotein-coding	-1.74	2.60E-04	3.27E-02	-2.69	4.89E-08	3.89E-05
AKR1C1I2-ALPHA-HSDI20-ALPHA-HSDIC9IDD1IDD1/DD2IDDHIDDH1IH-37IHAKRCIHBABIMBABI-I10p15-p14Iprotein-coding	-1.76	2.81E-04	3.39E-02	-2.75	5.12E-08	3.96E-05
PLAUIATFIBDPLT5IQPDIUPAIURKIu-PAI-I10q22.2Iprotein-coding	2.32	1.43E-06	4.47E-04	2.64	6.10E-08	4.45E-05
HIST1H2BDIH2B.1BIH2B/bIH2BFBIHIRIP2IdJ221C16.6I-I6p21.3Iprotein-coding	-1.99	6.75E-05	1.09E-02	-2.81	6.06E-08	4.45E-05
PDK4I-I-I7q21.3Iprotein-coding	-0.15	7.55E-01	1.00E+00	-2.88	7.00E-08	4.97E-05
LINC01497I-I-I17q24.3IncRNA	-3.49	2.15E-05	4.49E-03	-5.24	8.89E-08	6.15E-05
CCDC3I-I-I10p13Iprotein-coding	1.99	5.62E-05	9.64E-03	2.65	1.27E-07	8.52E-05
NTSR1INTRI-I20q13Iprotein-coding	1.71	5.25E-03	2.66E-01	3.09	1.31E-07	8.62E-05
SH2D5I-I-I1p36.12Iprotein-coding	1.16	2.33E-02	6.73E-01	2.70	1.57E-07	9.80E-05
HIST1H2ACIH2A/IIH2AFLIdJ221C16.4I-I6p22.1Iprotein-coding	-0.82	9.72E-02	1.00E+00	-2.87	1.57E-07	9.80E-05
INSCI-I-I11p15.2Iprotein-coding	2.96	1.12E-08	6.00E-06	2.69	1.72E-07	1.00E-04
TSPAN11IVSSW1971I-I12p11.21Iprotein-coding	-1.27	1.52E-02	5.25E-01	-3.01	1.70E-07	1.00E-04
CYP1B1ICP1BICYPIB1IGLC3AIP4501B1I-I2p22.2Iprotein-coding	-0.87	6.16E-02	1.00E+00	-2.56	1.65E-07	1.00E-04
DLL4Ihdelta2I-I15q14Iprotein-coding	2.29	2.41E-06	6.86E-04	2.55	1.96E-07	1.12E-04
LAMA2ILAMMI-I6q22-q23Iprotein-coding	-1.48	1.60E-03	1.21E-01	-2.51	2.54E-07	1.42E-04
APOL1IAPO-LIAPOLIAPOL-IIFSGS4I-I22q13.1Iprotein-coding	-1.14	3.08E-02	7.78E-01	-3.21	2.62E-07	1.43E-04
SLC40A1IFPN1IHFE4IIREG1IMST079IMSTP079IMTP1ISLC11A3I-I2q32Iprotein-coding	-1.37	4.01E-03	2.24E-01	-2.55	3.16E-07	1.70E-04
C11orf96IAG2I-I11p11.2Iprotein-coding	0.41	3.87E-01	1.00E+00	-2.74	3.58E-07	1.88E-04
LINC01503I-I-I9q34.11IncRNA	-1.67	2.10E-03	1.44E-01	-3.08	3.77E-07	1.94E-04

HEPG2 Cells **MS# GENESDEV/2016/280941_Franks et al._Supp. Figure 6**

Gene Name	Nup98 siRNA Log2 Fold Change	Nup98 siRNA P-Value	Nup98 siRNA Adjusted P-Value	sPom121 siRNA Log2 Fold Change	sPom121 siRNA P-Value	sPom121 siRNA Adjusted P-Value
GCIDBPIDBP/GCIGRD3IHEL-S-51IVDBGIVDBPI-I4q12-q13Iprotein-coding	-9.36	5.27E-38	1.38E-33	-8.57	5.78E-35	1.52E-30
NGFRICD271IGp80-LNGFRITNFRSF16Ip75(NTR)Ip75NTRI-I17q21-q22Iprotein-coding	3.02	8.53E-07	4.77E-04	5.41	3.44E-18	4.51E-14
IGFBP5IIBP5I-I2q35Iprotein-coding	-5.00	1.44E-01	1.00E+00	6.52	5.78E-18	5.06E-14
UGT2B15IHLUG4IUDPGT 2B8IUDPGT2B15IUDPGTH3IUGT2B8I-I4q13Iprotein-coding	-5.00	7.45E-17	9.78E-13	-4.38	4.67E-14	3.06E-10
HABP2IFSAPIHABPIHGFALIPHBPI-I10q25.3Iprotein-coding	-5.63	2.17E-15	1.43E-11	-4.72	1.64E-12	8.63E-09
EREGIERI-I4q13.3Iprotein-coding	5.69	3.06E-16	2.68E-12	4.93	2.39E-12	1.04E-08
CPLX2I921-LICPX-2ICPX2IHfb1I-I5q35.2Iprotein-coding	2.91	8.39E-09	9.70E-06	3.59	4.29E-12	1.61E-08
GGT5IGGLIGGT 5IGGT-RELIGGTLA1I-I22q11.23Iprotein-coding	3.55	1.45E-07	1.03E-04	4.56	6.83E-12	2.16E-08
SLC16A6IMCT6IMCT7I-I17q24.2Iprotein-coding	3.69	1.48E-11	3.90E-08	3.77	7.42E-12	2.16E-08
HMGCS2I-I1p13-p12Iprotein-coding	-1.98	5.64E-05	1.29E-02	-3.72	1.58E-11	4.15E-08
BEAN1IBEANISCA31I-I16q21Iprotein-coding	0.80	2.68E-01	1.00E+00	3.98	4.36E-11	1.04E-07
SETBP1IMRD29ISEBI-I18q21.1Iprotein-coding	-3.35	1.10E-09	1.80E-06	-3.80	5.48E-11	1.11E-07
VGLL3IVGL-3IVGL3I-I3p12.1Iprotein-coding	-1.90	1.28E-04	2.14E-02	-3.65	5.32E-11	1.11E-07
UGT2B11I-I4q13.2Iprotein-coding	-3.72	2.79E-12	9.16E-09	-3.45	6.30E-11	1.18E-07
PRF1IFLH2IHPLH2IP1IPFN1IPFPI-I10q22Iprotein-coding	2.35	6.33E-04	6.96E-02	4.23	9.24E-11	1.62E-07
CYP24A1ICP24ICYP24IHCAIIP450-CC24I-I20q13Iprotein-coding	2.92	9.98E-09	1.05E-05	3.25	3.09E-10	5.07E-07
SLC6A12IBGT-1IBGT1IGAT2I-I12p13Iprotein-coding	-1.67	4.19E-04	5.23E-02	-3.13	4.30E-10	6.64E-07
PLP1IGPM6CIHLD1IMMPLIPLPIPLP/DM20IPMDISPG2I-IXq22Iprotein-coding	5.99	1.79E-13	9.42E-10	5.14	5.09E-10	7.43E-07
C14orf105I-I-I14q22.3Iprotein-coding	-2.79	4.45E-07	2.66E-04	-3.82	7.44E-10	1.03E-06
LINC00324IC17orf44INCRNA00324I-I17p13.1IncRNA	-3.78	9.77E-12	2.85E-08	-3.30	1.70E-09	2.24E-06
ZBED6IMGRI-I1q32.1Iprotein-coding	-0.77	9.82E-02	1.00E+00	-3.06	2.94E-09	3.67E-06
KRT23ICK23IHAIK1IK23I-I17q21.2Iprotein-coding	1.86	1.21E-04	2.07E-02	2.93	4.92E-09	5.88E-06
SLC5A3IBCW2ISMITISMIT1ISMIT2I-I21q22.12Iprotein-coding	-2.10	1.13E-05	3.62E-03	-2.88	5.36E-09	5.94E-06
RIPPLY3IDSCR6I-I21q22.2Iprotein-coding	1.58	1.05E-02	4.41E-01	3.47	5.43E-09	5.94E-06
RBP1ICRABP-IICRBPICRBP1ICRBPIIRBPCI-I3q23Iprotein-coding	1.66	1.11E-03	1.04E-01	2.97	1.25E-08	1.31E-05
CDKL5IEIEE2IISSXISTK9I-IXp22Iprotein-coding	-0.09	8.77E-01	1.00E+00	-3.64	1.64E-08	1.65E-05
ETNPPLIAGXT2L1I-I4q25Iprotein-coding	-2.42	1.40E-06	6.82E-04	-2.90	2.39E-08	2.33E-05
SGK2IH-SGK2IdJ138B7.2I-I20q13.2Iprotein-coding	-2.32	2.22E-06	9.82E-04	-2.79	2.52E-08	2.37E-05
LYVE1ICRSBP-1IHARILYVE-1IXLKD1I-I11p15Iprotein-coding	0.17	7.90E-01	1.00E+00	2.95	2.81E-08	2.51E-05
KRTAP3-1IKAP3.1IKRTAP3.1I-I17q21.2Iprotein-coding	4.50	1.12E-07	8.41E-05	4.69	2.86E-08	2.51E-05
LINC00326INCRNA00326I-I6q23.2IncRNA	3.36	3.80E-04	4.99E-02	4.67	3.55E-08	3.00E-05
LOC101927884I-I-I2p12IncRNA	2.73	2.69E-05	7.14E-03	3.47	4.13E-08	3.39E-05
MT1GIMT1IMT1KI-I16q13Iprotein-coding	-2.62	2.65E-07	1.78E-04	-2.81	5.85E-08	4.65E-05
DIO1I5DIITXDI1I-I1p33-p32Iprotein-coding	-1.46	2.05E-03	1.52E-01	-2.71	6.09E-08	4.70E-05
CYP19A1IAROIARO1ICPV1ICYARICYP19ICYPXIXIP-450AROMI-I15q21.1Iprotein-coding	1.65	1.23E-03	1.14E-01	2.80	6.74E-08	5.06E-05
NR5A2IB1FIB1F2ICPFIFTFIFTZ-F1IFTZ-F1betaILRH-1ILRH1IhB1F-2I-I1q32.1Iprotein-coding	-2.41	6.20E-06	2.29E-03	-3.06	1.04E-07	7.38E-05
PON1IESAIMVCD5IPONI-I7q21.3Iprotein-coding	-4.18	6.76E-13	2.96E-09	-2.89	1.03E-07	7.38E-05
FAM160A1I-I-I4q31.3Iprotein-coding	-0.62	1.94E-01	1.00E+00	-2.89	1.20E-07	7.65E-05
MMP2ICLG4ICLG4AIMMP-2IMMP-IIIMONAITBE-1I-I16q13-q21Iprotein-coding	2.16	6.07E-05	1.34E-02	2.88	1.14E-07	7.65E-05
HRGIHPRGIHRGPITHPH11I-I3q27Iprotein-coding	-2.51	1.31E-05	3.96E-03	-3.48	1.17E-07	7.65E-05
LOC102724550I-I-I-IncRNA	-1.27	1.55E-02	5.45E-01	-3.38	1.13E-07	7.65E-05
MBOAT4IFKSG89IGOATIOACT4I-I8p12Iprotein-coding	-1.68	5.90E-04	6.56E-02	-2.76	1.24E-07	7.74E-05
ANXA13IANX13IISAI-I8q24.13Iprotein-coding	-2.58	9.11E-07	4.88E-04	-2.86	1.71E-07	1.04E-04
CACNG4I-I-I17q24Iprotein-coding	6.42	2.92E-03	1.92E-01	8.14	2.34E-07	1.35E-04
KLHDC7BI-I-I22q13.33Iprotein-coding	7.11	1.75E-04	2.76E-02	8.14	2.34E-07	1.35E-04
AMOTI-I-IXq23Iprotein-coding	-2.31	1.20E-04	2.07E-02	-3.78	2.37E-07	1.35E-04
CYP3A7-CYP3A51PICYP3A7-3AP1ICYP3A7-CYP3AP1ICYP3A7.1LI-I7q22.1Iprotein-coding	-2.05	2.36E-05	6.38E-03	-2.55	2.57E-07	1.44E-04
SLCO4C1IOATP-HIOATP-M1IOATP4C1IOATPXIPRO2176ISLC21A20I-I5q21.2Iprotein-coding	-1.05	2.47E-02	6.81E-01	-2.54	2.71E-07	1.48E-04
LCN15IPRO6093IUNQ2541I-I9q34.3Iprotein-coding	0.53	3.04E-01	1.00E+00	2.64	2.82E-07	1.51E-04
EDN1IARCND3IET1IHDLCQ7IPPET1IQMEI-I6p24.1Iprotein-coding	3.36	8.49E-09	9.70E-06	3.04	3.55E-07	1.86E-04

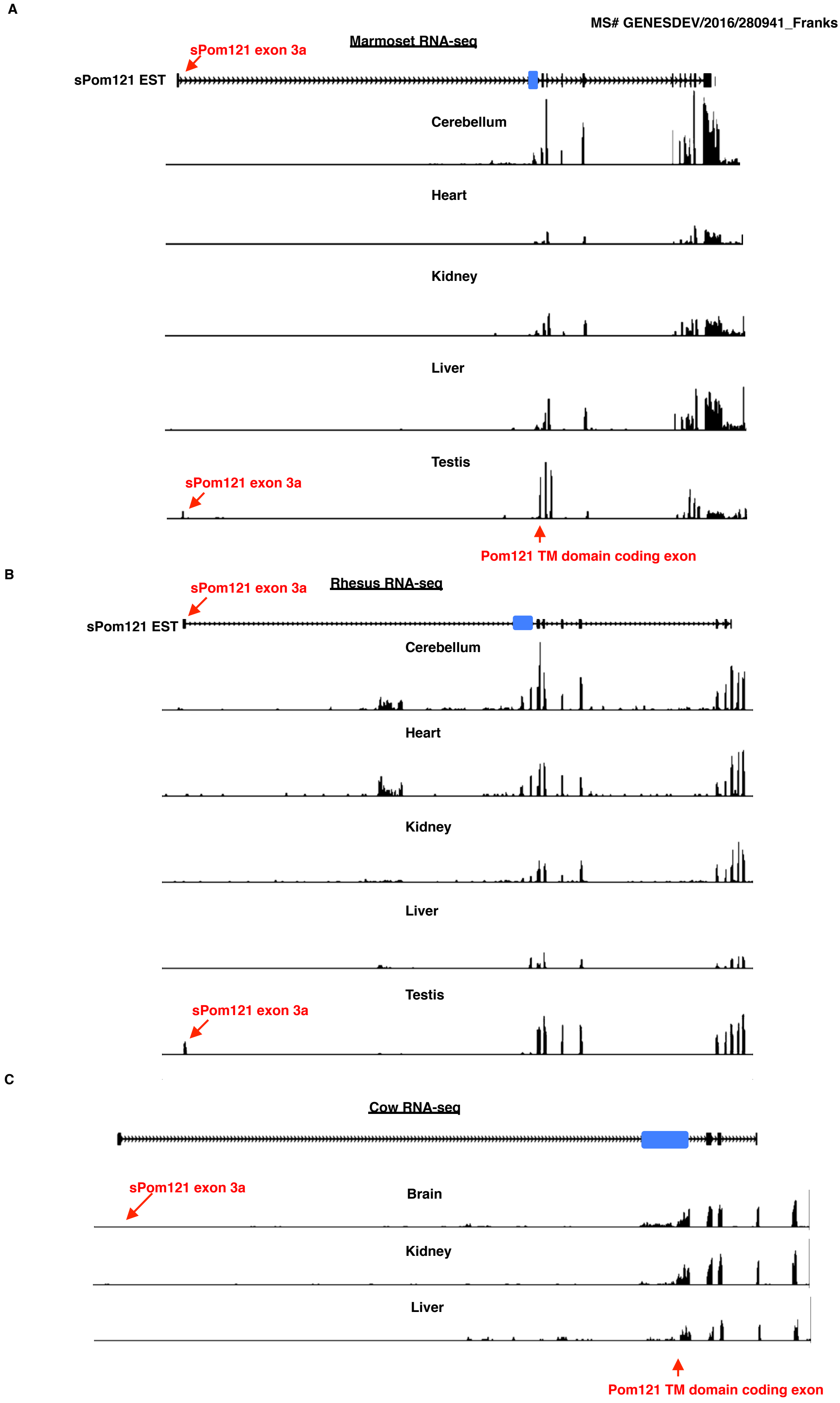
A
Marmoset RNA-seq
sPom121 exon 3a
sPom121 EST
Cerebellum
Heart
Kidney
Liver
Testis
sPom121 exon 3a
Pom121 TM domain coding exon
B
Rhesus RNA-seq
sPom121 exon 3a
sPom121 EST
Cerebellum
Heart
Kidney
Liver
Testis
sPom121 exon 3a
C
Cow RNA-seq
sPom121 exon 3a
Brain
Kidney
Liver
Pom121 TM domain coding exon
